# Supplementary material for: Development and validation of a life expectancy estimator for multimorbid older adults: a cohort study protocol
Source: BMJ Open. 2021 Aug 25;11(8):e048168. doi: 10.1136/bmjopen-2020-048168 (PMC8388271; doi:10.1136/bmjopen-2020-048168)
Supplement: Author's manuscript [file bmjopen-2020-048168.draft_revisions.pdf]

BMJ Open

BMJ Open is committed to open peer review. As part of this commitment we make the peer review history of every article we publish publicly available.

When an article is published we post the peer reviewers' comments and the authors' responses online. We also post the versions of the paper that were used during peer review. These are the versions that the peer review comments apply to.

The versions of the paper that follow are the versions that were submitted during the peer review process. They are not the versions of record or the final published versions. They should not be cited or distributed as the published version of this manuscript.

BMJ Open is an open access journal and the full, final, typeset and author-corrected version of record of the manuscript is available on our site with no access controls, subscription charges or pay-per-view fees (http://bmjopen.bmj.com).

If you have any questions on BMJ Open's open peer review process please email info.bmjopen@bmj.com

BMJ Open

# Development and validation of a life expectancy estimator for multimorbid older adults: a cohort study protocol

| | |
|---|---|
| Journal: | *BMJ Open* |
| Manuscript ID | bmjopen-2020-048168 |
| Article Type: | Protocol |
| Date Submitted by the Author: | 18-Dec-2020 |
| Complete List of Authors: | Gastens, Viktoria; University of Bern, Institute of Primary Health Care (BIHAM); University of Bern, Graduate School for Health Sciences<br>Del Giovane, Cinzia; University of Bern, Institute of Primary Health Care (BIHAM); University of Fribourg, Population Health Laboratory (#PopHealthLab)<br>Anker, Daniela; University of Bern, Institute of Primary Health Care (BIHAM); University of Fribourg, Population Health Laboratory (#PopHealthLab),<br>Feller, Martin; University of Bern, Institute of Primary Health Care (BIHAM); University of Bern, Department of General Internal Medicine, Inselspital, Bern University Hospital<br>Syrogiannouli, Lamprini; University of Bern, Institute of Primary Health Care (BIHAM)<br>Schwab, Nathalie; University of Bern, Department of General Internal Medicine, Inselspital, Bern University Hospital<br>Bauer, Douglas; University of California San Francisco, Departments of Medicine and Epidemiology & Biostatistics<br>Rodondi, Nicolas; University of Bern, Institute of Primary Health Care (BIHAM); University of Bern, Department of General Internal Medicine, Inselspital, Bern University Hospital<br>Chiolero, Arnaud; University of Fribourg, Population Health Laboratory (#PopHealthLab); McGill University, School of Global and Population Health |
| Keywords: | PREVENTIVE MEDICINE, PUBLIC HEALTH, STATISTICS & RESEARCH METHODS |
| | |

SCHOLARONE™
Manuscripts

*I, the Submitting Author has the right to grant and does grant on behalf of all authors of the Work (as defined in the below author licence), an exclusive licence and/or a non-exclusive licence for contributions from authors who are: i) UK Crown employees; ii) where BMJ has agreed a CC-BY licence shall apply, and/or iii) in accordance with the terms applicable for US Federal Government officers or employees acting as part of their official duties; on a worldwide, perpetual, irrevocable, royalty-free basis to BMJ Publishing Group Ltd ("BMJ") its licensees and where the relevant Journal is co-owned by BMJ to the co-owners of the Journal, to publish the Work in this journal and any other BMJ products and to exploit all rights, as set out in our licence.*

*The Submitting Author accepts and understands that any supply made under these terms is made by BMJ to the Submitting Author unless you are acting as an employee on behalf of your employer or a postgraduate student of an affiliated institution which is paying any applicable article publishing charge ("APC") for Open Access articles. Where the Submitting Author wishes to make the Work available on an Open Access basis (and intends to pay the relevant APC), the terms of reuse of such Open Access shall be governed by a Creative Commons licence – details of these licences and which Creative Commons licence will apply to this Work are set out in our licence referred to above.*

*Other than as permitted in any relevant BMJ Author's Self Archiving Policies, I confirm this Work has not been accepted for publication elsewhere, is not being considered for publication elsewhere and does not duplicate material already published. I confirm all authors consent to publication of this Work and authorise the granting of this licence.*

For peer review only

# Development and validation of a life expectancy estimator for multimorbid older adults: a cohort study protocol

Viktoria Gastens[1,2], Cinzia Del Giovane[1,5], Daniela Anker[1,5], Martin Feller[1,3], Lamprini Syrogiannouli[1], Nathalie Schwab[1,3], Douglas C. Bauer[6], Nicolas Rodondi[1,3], and Arnaud Chiolero[1,4,5]

[1]Institute of Primary Health Care (BIHAM), University of Bern, Bern, Switzerland
[2]Graduate School for Health Sciences, University of Bern, Bern, Switzerland
[3]Department of General Internal Medicine, Inselspital, Bern University Hospital, University of Bern, Bern, Switzerland
[4]School of Global and Population Health, McGill University, Montreal, Canada
[5]Population Health Laboratory (#PopHealthLab), Department of Community Health, University of Fribourg, Fribourg, Switzerland
[6]Departments of Medicine and Epidemiology & Biostatistics, University of California, San Francisco, CA, USA

October 20, 2020

**Correspondence to**
Viktoria Gastens
Institute of Primary Health Care (BIHAM)
University of Bern
Mittelstrasse 43
3012 Bern, Switzerland
> viktoria.gastens@biham.unibe.ch

**Word count**
2000 / 4000

For peer review only



For peer review only - http://bmjopen.bmj.com/site/about/guidelines.xhtml

## ABSTRACT

### Introduction

Older multimorbid adults have a high risk of mortality and short life expectancy (LE). Providing high-value care and avoiding care overuse, including of preventive care, is a serious challenge among multimorbid patients. Although guidelines recommend to tailor preventive care according to the estimated LE, a LE estimator does not exist for this specific population. Our objective is therefore to develop a LE estimator for older multimorbid adults by transforming a mortality prognostic index, which will be developed and internally validated in a prospective cohort.

### Methods and analysis

We will analyse data of the OPERAM cohort study in Bern, Switzerland. 822 participants were included at hospitalisation with age of 70 years or older, multimorbidity (3 or more chronic medical conditions) and polypharmacy (use of 5 drugs or more for >30 days). All-cause mortality will be assessed during 3 years of follow-up. We will perform a Cox regression model with backward stepwise selection to identify the mortality risk predictors. We will use the Gronnesby and Borgan test to assess model calibration and the Harrell's C statistic for model discrimination. The model will be internally validated using bootstrapping techniques. We will derive a point-based risk score from the regression coefficients. We will transform the 3-year mortality prognostic index into a LE estimator using the Gompertz survival function. We will perform a qualitative assessment of the clinical usability of the LE estimator and its application. We will conduct the development and validation of the mortality prognostic index following the Prognosis Research Strategy (PROGRESS) framework and report it following the Transparent Reporting of a multivariable prediction model for Individual Prognosis Or Diagnosis (TRIPOD) statement.

### Ethics and dissemination

Written informed consent by patients themselves or, in the case of cognitive impairment by a legal representative, was required before enrolment. The local ethics committee has approved the study.


## STRENGTHS AND LIMITATIONS OF THIS STUDY

- We will provide the first life expectancy estimator specifically for older multimorbid adults.
- We use high-quality data from a large prospective cohort study of multimorbid older adults.
- This predictive tool may help clinicians to personalize preventive care according to individual life expectancy.
- External validation of the new life expectancy estimator in another cohort will still be required.



For peer review only - http://bmjopen.bmj.com/site/about/guidelines.xhtml

## INTRODUCTION

Multimorbidity is frequent among older adults and is associated with shortened life expectancy (LE),[1] low quality of life, and high health care utilisation such as polypharmacy [2] and multiple hospital admissions.[3] While the benefits and harms of preventive care, such as cancer screening and cardiovascular disease (CVD) preventive treatment, are well known up to the age of 75 years, they are not directly applicable to older multimorbid patients.[6, 7] For this reason, international guidelines such as USPSTF (U.S. Preventive Services Task Force) recommend accounting for LE when deciding upon preventive care.

Accounting for LE is indeed key because most preventive care has a lagtime to benefit. The lagtime to benefit is the time interval between an intervention (e.g., cancer screening) and the moment when health outcomes are improved (e.g., lower mortality). It varies greatly, ranging from 6 months for statin treatment in the secondary prevention of CVD to up to 10 years for several cancer screenings.[9, 10] If the lagtime to benefit of the intervention is longer than the estimated LE, the patient cannot benefit from it [11, 12] and the intervention should not be recommended. Older multimorbid adults are at high risk of not having the time to benefit, and harms may outweigh the benefits of an intervention. To decide which preventive care should be delivered, the lagtime to benefit should be therefore weighted against the estimated LE, as proposed for cancer screening by Lee et al.[9, 10]

There is however no tool to accurately estimate LE among multimorbid patients. LE estimators are usually derived from mortality risk scores. Several risk scores allow estimating mortality risk for older adults,[11] but they are not recommended for widespread use and do not directly translate to LE estimates. To our knowledge, only Lee et al. have developed an LE estimator from a mortality risk prognostic index from a cohort of community dwelling older adults with a relatively low mortality rate,[12] but it is not directly applicable to multimorbid patients and other populations with relatively high mortality rates. Our objective is therefore to develop and internally validate a LE estimator for older multimorbid adults.



For peer review only - http://bmjopen.bmj.com/site/about/guidelines.xhtml

## METHODS AND ANALYSIS

### Source of data and study design

We will use data from 822 participants of an ongoing study in Bern, Switzerland. Participants were originally enrolled in a clinical trial with an intervention lasting 1 year and will be followed-up post-intervention for an additional two years as a cohort. The clinical trial was the cluster randomized controlled trial OPtimising thERapy to prevent Avoidable hospital admissions in Multimorbid older people (OPERAM; [13]). Participants were assigned to receive either standard care or a medication review by a Systematic Tool to Reduce Inappropriate Prescribing (STRIP) with observation of the primary outcome of drug-related hospital admission (DRA) over 1 year. For our study, we will use data collected at baseline (December 2016-October 2018) and up to 3 years after baseline (until October 2021). **Figure 1** shows the schedule of data collection for this project.

**Figure 1.** Schedule of data collection from baseline to 3-year follow-up.

Inclusion:
- ≥70 years
- Multimorbidity (≥3 conditions)
- Polypharmacy (≥5 drugs)

Exclusion:
- Admission to palliative care (<24 h)
- Structured drug review (<2 months)
- No informed consent

| | **Baseline** First patient first visit 12/2016 | **1 year follow-up** | **2 year follow-up** | **3 year follow-up** Last patient last visit 10/2021 |
|---|---|---|---|---|
| Baseline characteristics | X | | | |
| Activities of Daily Living (ADL) | X | X | | |
| Quality of Life (EQ-5D) | X | X | | |
| Falls and fractures | X | X | | |
| Health economics | X | X | | |
| Diagnoses | X | X | X | X |
| Medication | X | X | X | X |
| Blood pressure and cholesterol | X | X | X | X |
| Life expectancy questionnaire by Lee et al.[1] | | X | X | X |
| Cancer screening | | X | X | X |

[1] SJ Lee et al. "Development and Validation of a Prognostic Index for 4-Year Mortality in Older Adults". *JAMA* 2006

Study nurses collected baseline data by a personal interview with the participant and from medical files. Follow-ups are conducted via phone calls. Phone interviews are held with participants or relatives, otherwise with a proxy or with the general practitioner, when the participants are not reachable or not able to answer.

### Participants

822 participants were enrolled at hospitalisation in the Inselspital, University Hospital, Bern, Switzerland. Inclusion criteria were age of 70 years or older, multimorbidity (3 or more chronic medical conditions) and polypharmacy (use of 5 drugs or more for >30 days). A detailed account of the study participants' baseline characteristics is given in **Table 1**.



For peer review only - http://bmjopen.bmj.com/site/about/guidelines.xhtml

**Table 1.** Baseline characteristics of participants. IQR: interquartile range; BMI: body mass index; CVD: cardiovascular disease.

| Baseline characteristic | n (%) |
|---|---|
| N | 822 |
| Female | 347 (42.2) |
| Age, median [IQR] | 79 [74.0; 84.0] |
| BMI, median [IQR] | 26.0 [23.0; 29.1] |
| Smoking | 69 (8.4) |
| Education | |
| University | 151 (18.4) |
| High school | 491 (59.7) |
| Less than high school | 170 (20.7) |
| Dementia | 82 (10.0) |
| History of Cancer | 324 (39.4) |
| History of CVD | 528 (64.2) |
| CVD risk factors and medication | |
| Hypertension | 626 (76.2) |
| Hypertension with treatment | 529 (64.4) |
| Hypercholesterolemia | 309 (37.6) |
| Hypercholesterolemia with treatment | 229 (27.9) |
| Diabetes | 256 (31.1) |
| Diabetes with treatment | 202 (24.6) |
| Chronic medication, median [IQR] | 10 [6.5; 13.5] |
| Diagnoses, median [IQR] | 16 [12.5; 19.5] |
| Hospitalisations during the last year, median [IQR] | 1 [0.5; 1.5] |

Written informed consent by patients themselves or, in the case of cognitive impairment by a legal representative, had already been obtained before enrolment. Patients planned for direct admission to palliative care (<24 hours after admission), or patients undergoing a structured drug review other than the trial intervention, or who had passed a structured drug review within the last 2 months were excluded. Also patients for whom it was not possible to obtain an informed consent were excluded.

## Outcome

The outcome of interest is all-cause mortality over the 3 years of follow-up. 153 deaths (18.6%) occurred over the first year of follow-up. Since participants were recruited during hospital admission, a higher mortality rate is expected during the first year of follow-up compared with subsequent follow-up and we expect approximately 250 deaths (30%) over the 3 years of follow-up.

## Development, validation, and clinical usability of a life expectancy estimator for multimorbid adults

We will develop and validate the mortality prognostic index following the Prognosis Research Strategy (PROGRESS) framework,[14] and report it following the Transparent Reporting of a multivariable prediction model for Individual Prognosis Or Diagnosis (TRIPOD) statement.[15] We will further follow the recommendations of Moons et al. [16, 17] for risk prediction models and apply the method described below for 3-year mortality prediction models. **Figure 2** summarizes the process of developing the life expectancy estimator tool.



For peer review only - http://bmjopen.bmj.com/site/about/guidelines.xhtml

**Figure 2.** Flowchart of the development steps (boxes) and methods used (arrows) for the life expectancy estimator tool.

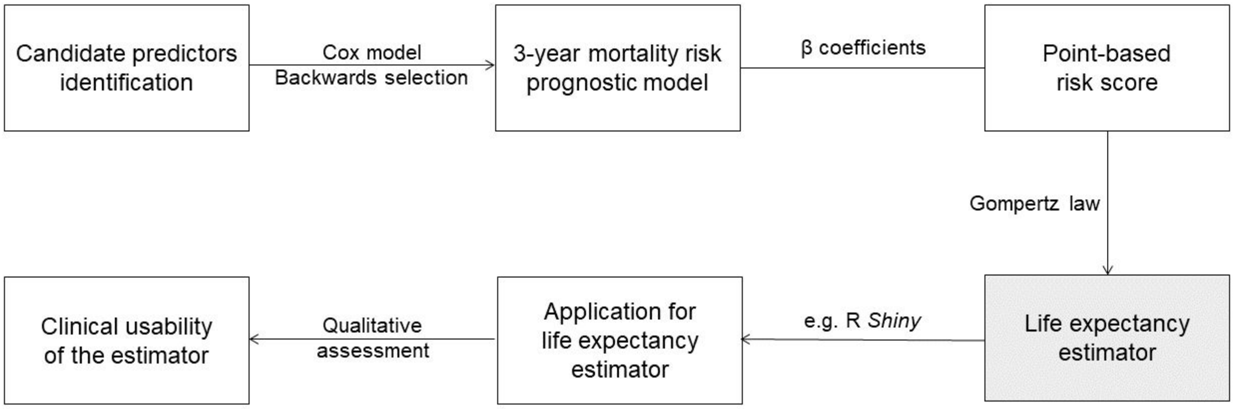


*Candidate predictors identification*

Candidate predictors are usually derived from previous research efforts in this field,[11, 18] ease and reliability of measurement in clinical setting, and background knowledge on potential associations with the outcome. Therefore, we will perform a literature review of potential predictors, and also consider factors included in the OPERAM dataset that may not be identified from the review but specific to the target population. **Table 2** shows a list of potential candidate predictors. They include demographic (age, sex), clinical characteristics (comorbidity, medication, body mass index), smoking, functional status variables, and hospitalization. Notably, we will account for the severity of comorbidity and functional impairment, which has not been considered by Lee et al.[12]

**Table 2.** Potential candidate predictors for the Cox regression analysis from information obtained at baseline.

| Variable | Description |
|---|---|
| Age | |
| Sex | |
| Comorbidities | Charlson Comorbidity Index (CCI) |
| Medication burden | Number of drugs before index hospitalization |
| Body-Mass-Index (BMI) | Ratio of weight (kg) / square of height ($m^2$) |
| Weight loss | Lost weight during last year (yes/no) |
| Smoking | Current smoker (yes/no) |
| Hospitalisation | Number of hospitalisations during last year |
| Activities of Daily Living (ADL) | Barthel-Index: bathing, bladder control, bowel control, dressing, feeding, grooming, mobility, stairs, toilet use, transfer |
| Generic health status | EQ-5D score: mobility, self-care, usual activities, pain/discomfort and anxiety/depression |
| Falls | Number of falls during last year |
| Nursing | Nursing home residency (yes/no) |

As part of the OPERAM trial, the participants have been assigned to a control or intervention group at baseline. We will test the predictive effect of this assignment on mortality and account for it in the analyses if necessary.



For peer review only - http://bmjopen.bmj.com/site/about/guidelines.xhtml

*Mortality risk prognostic model*

The relationship between candidate predictors and outcome will be analysed using multivariable Cox regression. We will use the backward selection method and remove candidates with a p-value >0.05.[16, 17] The remaining variables will form the final model. We will use multiple imputation for missing values under a missing at random assumption in order to reduce bias and avoid excluding participants from the analysis.[16] We will investigate the predictive accuracy of the final model by testing calibration and discrimination. We will assess the model's calibration by graphical calibration plots and by comparing the predicted to the observed mortality, with a relative difference of less than 10% considered satisfactory.[11] In addition, we will examine how closely the model's predictions match observed mortality rates for various risk groups of participants by Gronnesby and Borgan test.[19] The model's discrimination will be assessed with Harrell's C statistic.[11] We will evaluate potential overfitting and optimism by internal validation with bootstrapping techniques.[16, 17] If necessary, we will adjust the model for overfitting.

We have calculated the required sample size for conducting our multivariable prediction model utilizing the criteria proposed by Riley et al. [20] and implemented in the *pmsampsize* library for the R environment.[21] For the 3-year mortality risk prognostic model, the minimum sample size required with 12 candidate predictor parameters, an expected outcome event rate of 0.1 per year, and an anticipated Cox-Snell $R^2$ 0.126 (C statistic of [18] 0.82 [18, 22]) is 799 with 20 events per predictor parameter. This is considerably more than the idiomatic 10 events per predictor parameter. Our sample size of 822 is therefore adequate for this project.

*Point-based risk score*

From the final model, we will derive a point-based risk score by assigning points to each risk factor. Each $\beta$ coefficient will be divided by the lowest $\beta$ coefficient and rounded to the nearest integer. The sum of points for each risk factor will then represent the total risk score of this participant.[18]

*Life expectancy estimator*

We will transform the 3-year prognostic index into a LE estimator following the method of Lee et al.[12] In particular, we will use the new 3-year mortality prognostic index to define subpopulations with the same risk score.[12] We will fit a Gompertz survival function with each point score as a categorical predictor having a flexible proportional effect on the hazard rate. The Gompertz function assumes that each subpopulation will experience an exponential rise in mortality risk over time ($h_i(t) = \lambda_i \exp(\gamma t)$ , where $\lambda_i = \exp(x_i\beta)$). The Gompertz model will allow us to determine the time to 25%, 50% and 75% mortality for each of the risk point groups. We will report 95% confidence for the median survival intervals using bootstrapping techniques as well as 50% prediction intervals. We will compare our fit Gompertz model with observed Kaplan-Meier (KM) survival curves. Model calibration will again be assessed using the Hosmer-Lemeshow test [23] and model discrimination using Harrell's C statistic. The Lee LE estimator [12] will be recalibrated using standard methods to reflect the higher mortality rates of the target population.[17] The performance of the new LE estimator will be compared to the recalibrated Lee LE estimator by the Brier score [24] and Harrell's C statistic. We will build an interactive web application of the final model using the Shiny package in the R environment.



For peer review only - http://bmjopen.bmj.com/site/about/guidelines.xhtml

*Clinical usability of the new LE estimator*

We will perform a qualitative assessment of the LE estimator with one-to-one interviews with at least 10 clinicians.[25] We will assess whether the prediction parameters are pertinent and measurable in clinical practice, and evaluate barriers and enablers for impactful implementation of the tool. If no consent on the parameters to be included is found, we will use an iterative process, based on the Delphi-method.[26] Identified barriers and enablers will be used for the elaboration of implementation strategies.

## CONCLUSION

To our knowledge, we will be the first to develop an LE estimator specifically for older multimorbid adults. A major strength of this study is its high feasibility due to the follow-up of 822 already included multimorbid participants with strong strategies of containment of loss to follow-up. Notably, individuals with cognitive impairment were included, who are commonly excluded from studies. In addition to the development and internal validation of the LE estimator, we also assess its implementation and clinical usability. One major limitation is the lack of external validation but we will explore opportunities to test the LE estimator in a different dataset of older multimorbid adults. Our results will be useful for both clinical and research activities as they can have a major impact on preventive care practice by helping health care providers to tailor preventive care according to the estimated LE. Eventually, our study will help preventing under- and overuse of preventive care in the growing older population.

For peer review only



For peer review only - http://bmjopen.bmj.com/site/about/guidelines.xhtml

## REFERENCES


[1] Alessandro Menotti et al. “Prevalence of morbidity and multimorbidity in elderly male populations and their impact on 10-year all-cause mortality: The FINE study (Finland, Italy, Netherlands, Elderly)”. In: *Journal of clinical epidemiology* 54.7 (2001), pp. 680–686. doi: 10.1016/s0895-4356(00)00368-1.

[2] Carole E Aubert et al. “Polypharmacy and specific comorbidities in university primary care settings”. In: *European journal of internal medicine* 35 (2016), pp. 35–42. doi: 10.1016/j.ejim.2016.05.022.

[3] Anna Cassell et al. “The epidemiology of multimorbidity in primary care: a retrospective cohort study”. In: *Br J Gen Pract* 68.669 (2018), e245–e251. doi: 10.3399/bjgp18X695465.

[4] Tammy C. Hoffmann and Chris Del Mar. “Patients’ Expectations of the Benefits and Harms of Treatments, Screening, and Tests: A Systematic Review ”. In: *JAMA Internal Medicine* 175.2 (Feb. 2015), pp. 274–286. doi: 10.001/jamainternmed.2014.6016.

[5] Tammy C. Hoffmann and Chris Del Mar. “Clinicians’ Expectations of the Benefits and Harms of Treatments, Screening, and Tests: A Systematic Review”. In: *JAMA Internal Medicine* 177.3 (Mar. 2017), pp. 407–419. doi: 10.1001/jamainternmed.2016.8254.

[6] Janice B. Schwartz. “Primary prevention: Do the very elderly require a different approach?” In: *Trends in Cardiovascular Medicine* 25.3 (2015), pp. 228–239. doi: 10.1016/j.tcm.2014.10.010.

[7] Iris Lansdorp-Vogelaar et al. “Personalizing age of cancer screening cessation based on comorbid con- ditions: model estimates of harms and benefits”. In: *Annals of internal medicine* 161.2 (2014), pp. 104– 112. doi: 10.7326/M13-2867.

[8] David Blumenthal and Melinda K. Abrams. “Tailoring Complex Care Management for High-Need, High-Cost Patients”. In: *JAMA* 316.16 (Oct. 2016), pp. 1657–1658. doi: 10.1001/jama.2016.12388.

[9] Sei J Lee et al. “Time lag to benefit after screening for breast and colorectal cancer: meta-analysis of survival data from the United States, Sweden, United Kingdom, and Denmark”. In: *BMJ* 346 (2013). doi: 10.1136/bmj.e8441.

[10] Sei J. Lee, Rosanne M. Leipzig, and Louise C. Walter. “Incorporating Lag Time to Benefit Into Prevention Decisions for Older Adults”. In: *JAMA* 310.24 (Dec. 2013), pp. 2609–2610. doi: 10.1001/jama.2013.282612.

[11] Lindsey C. Yourman et al. “Prognostic Indices for Older Adults: A Systematic Review”. In: *JAMA* 307.2 (Jan. 2012), pp. 182–192. doi: 10.1001/jama.2011.1966.

[12] Sei J. Lee et al. “Individualizing Life Expectancy Estimates for Older Adults Using the Gompertz Law of Human Mortality”. In: *PLOS ONE* 9.9 (Sept. 2014), pp. 1–8. doi: 10.1371/journal.pone.0108540.

[13] Luise Adam et al. “Rationale and design of OPtimising thERapy to prevent Avoidable hospital admis- sions in Multimorbid older people (OPERAM): a cluster randomised controlled trial”. In: *BMJ Open* 9.6 (2019). doi: 10.1136/bmjopen-2018-026769.

[14] Ewout W Steyerberg et al. “Prognosis Research Strategy (PROGRESS) 3: prognostic model research”. In: *PLoS medicine* 10.2 (2013). doi: 10.1371/journal.pmed.1001381.

[15] Gary S Collins et al. “Transparent reporting of a multivariable prediction model for individual prognosis or diagnosis (TRIPOD): the TRIPOD statement”. In: *BMJ* 350 (2015). doi: 10.1136/bmj.g7594.

[16] Karel G M Moons et al. “Risk prediction models: I. Development, internal validation, and assessing the incremental value of a new (bio)marker”. In: *Heart* 98.9 (2012), pp. 683–690. doi: 10.1136/heartjnl-2011-301246.

[17] Karel G M Moons et al. “Risk prediction models: II. External validation, model updating, and impact assessment”. In: *Heart* 98.9 (2012), pp. 691–698. doi: 10.1136/heartjnl-2011-301247.

[18] Sei J. Lee et al. “Development and Validation of a Prognostic Index for 4-Year Mortality in Older Adults”. In: *JAMA* 295.7 (Feb. 2006), pp. 801–808. doi: 10.1001/jama.295.7.801.

[19] Grønnesby, Jon Ketil, and Ørnulf Borgan. "A method for checking regression models in survival analysis based on the risk score." *Lifetime data analysis* 2.4 (1996): 315-328.

[20] Richard D Riley et al. “Calculating the sample size required for developing a clinical prediction model”. In: *BMJ* 368 (2020). doi: 10.1136/bmj.m441.

[21] Joie Ensor, Emma C. Martin, and Richard D. Riley. *pmsampsize: Calculates the Minimum Sample Size Required for Developing a Multivariable Prediction Model*. R package version 1.0.2 — For new features, see the ’Changelog’ file (in the package source). 2019.

[22] Richard D Riley et al. “Minimum sample size for developing a multivariable prediction model: PART II - binary and time-to-event outcomes”. In: *Statistics in Medicine* 38.7 (2019), pp. 1276–1296. doi: 10.1002/sim.7992.

[23] Stanley Lemeshow and David W Hosmer Jr. “A review of goodness of fit statistics for use in the development of logistic regression models”. In: *American journal of epidemiology* 115.1 (1982), pp. 92– 106. doi: 10.1093/oxfordjournals.aje.a113284.




For peer review only - http://bmjopen.bmj.com/site/about/guidelines.xhtml

[24] Nancy R. Cook. “Use and Misuse of the Receiver Operating Characteristic Curve in Risk Prediction”. In: *Circulation* 115.7 (2007), pp. 928–935. doi: 10.1161/CIRCULATIONAHA.106.672402.
[25] Rosanna L Breen. “A practical guide to focus-group research”. In: *Journal of Geography in Higher Education* 30.3 (2006), pp. 463–475. doi: 10.1080/03098260600927575.
[26] RAND Corporation. *Delphi Method*. url: https://www.rand.org/topics/delphi-method.html.

## Authors’ contributions

Study concept and design: AC, NR, CDG. Acquisition, analysis, interpretation of data: NS, VG, CDG, LS. Drafting of the article: VG, CDG, AC. Critical revision of the article: all authors. Statistical analysis: VG, LS, CDG. Obtained funding: AC. Administrative, technical or material support: NS. Supervision: AC, CDG.

## Funding

This work was supported by the Swiss National Science Foundation grant number (320030_188549/01 to Prof Chiolero). This work is part of the project “OPERAM: OPtimising thERapy to prevent Avoidable hospital admissions in the Multimorbid elderly” supported by the European Union's Horizon 2020 research and innovation programme under the grant agreement No 6342388, and by the Swiss State Secretariat for Education, Research and Innovation (SERI) under contract number 15.0137. The opinions expressed and arguments employed herein are those of the authors and do not necessarily reflect the official views of the EC and the Swiss government.

## Competing interests

None declared.

## Ethics approval

The local Ethics Committee in Bern, Switzerland, approved the study protocol with the project number 2018-00784.

For peer review only

Inclusion:
- ≥70 years
- Multimorbidity (≥3 conditions)
- Polypharmacy (≥5 drugs)

Exclusion:
- Admission to palliative care (<24 h)
- Structured drug review (<2 months)
- No informed consent

| | **Baseline** First patient first visit 12/2016 | **1 year follow-up** | **2 year follow-up** | **3 year follow-up** Last patient last visit 10/2021 |
|---|---|---|---|---|
| Baseline characteristics | X | | | |
| Activities of Daily Living (ADL) | X | X | | |
| Quality of Life (EQ-5D) | X | X | | |
| Falls and fractures | X | X | | |
| Health economics | X | X | | |
| Diagnoses | X | X | X | X |
| Medication | X | X | X | X |
| Blood pressure and cholesterol | X | X | X | X |
| Life expectancy questionnaire by Lee et al.[1] | | X | X | X |
| Cancer screening | | X | X | X |

[1] SJ Lee et al. "Development and Validation of a Prognostic Index for 4-Year Mortality in Older Adults". *JAMA* 2006

Schedule of data collection from baseline to 3-year follow-up.

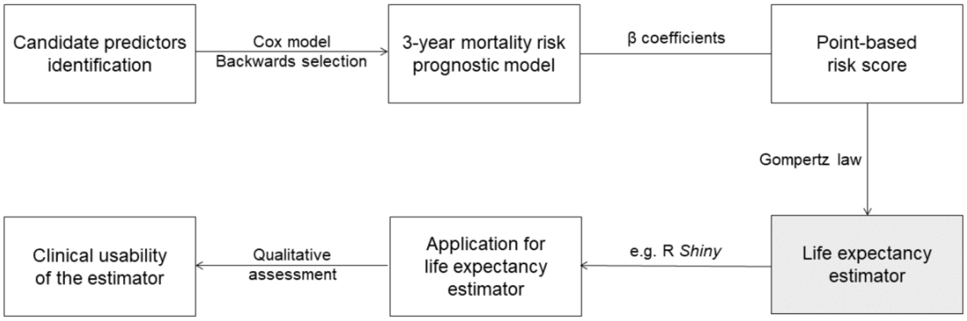


Flowchart of the development steps (boxes) and methods used (arrows) for the life expectancy estimator tool.

# TRIPOD Checklist: Prediction Model Development and Validation

| Section/Topic | Item | | Checklist Item | Page |
|---|---|---|---|---|
| **Title and abstract** | | | | |
| Title | 1 | D;V | Identify the study as developing and/or validating a multivariable prediction model, the target population, and the outcome to be predicted. | 1 |
| Abstract | 2 | D;V | Provide a summary of objectives, study design, setting, participants, sample size, predictors, outcome, statistical analysis, results, and conclusions. | 2 |
| **Introduction** | | | | |
| Background and objectives | 3a | D;V | Explain the medical context (including whether diagnostic or prognostic) and rationale for developing or validating the multivariable prediction model, including references to existing models. | 3 |
| | 3b | D;V | Specify the objectives, including whether the study describes the development or validation of the model or both. | 3 |
| **Methods** | | | | |
| Source of data | 4a | D;V | Describe the study design or source of data (e.g., randomized trial, cohort, or registry data), separately for the development and validation data sets, if applicable. | 4 |
| | 4b | D;V | Specify the key study dates, including start of accrual; end of accrual; and, if applicable, end of follow-up. | 4 |
| Participants | 5a | D;V | Specify key elements of the study setting (e.g., primary care, secondary care, general population) including number and location of centres. | 4 |
| | 5b | D;V | Describe eligibility criteria for participants. | 4 |
| | 5c | D;V | Give details of treatments received, if relevant. | 4 |
| Outcome | 6a | D;V | Clearly define the outcome that is predicted by the prediction model, including how and when assessed. | 5 |
| | 6b | D;V | Report any actions to blind assessment of the outcome to be predicted. | NA |
| Predictors | 7a | D;V | Clearly define all predictors used in developing or validating the multivariable prediction model, including how and when they were measured. | 6 |
| | 7b | D;V | Report any actions to blind assessment of predictors for the outcome and other predictors. | NA |
| Sample size | 8 | D;V | Explain how the study size was arrived at. | 7 |
| Missing data | 9 | D;V | Describe how missing data were handled (e.g., complete-case analysis, single imputation, multiple imputation) with details of any imputation method. | 7 |
| Statistical analysis methods | 10a | D | Describe how predictors were handled in the analyses. | 7 |
| | 10b | D | Specify type of model, all model-building procedures (including any predictor selection), and method for internal validation. | 7 |
| | 10c | V | For validation, describe how the predictions were calculated. | 7 |
| | 10d | D;V | Specify all measures used to assess model performance and, if relevant, to compare multiple models. | 7 |
| | 10e | V | Describe any model updating (e.g., recalibration) arising from the validation, if done. | 7 |
| Risk groups | 11 | D;V | Provide details on how risk groups were created, if done. | 7 |
| Development vs. validation | 12 | V | For validation, identify any differences from the development data in setting, eligibility criteria, outcome, and predictors. | NA |
| **Results** | | | | |
| Participants | 13a | D;V | Describe the flow of participants through the study, including the number of participants with and without the outcome and, if applicable, a summary of the follow-up time. A diagram may be helpful. | NA |
| | 13b | D;V | Describe the characteristics of the participants (basic demographics, clinical features, available predictors), including the number of participants with missing data for predictors and outcome. | NA |
| | 13c | V | For validation, show a comparison with the development data of the distribution of important variables (demographics, predictors and outcome). | NA |
| Model development | 14a | D | Specify the number of participants and outcome events in each analysis. | NA |
| | 14b | D | If done, report the unadjusted association between each candidate predictor and outcome. | NA |
| Model specification | 15a | D | Present the full prediction model to allow predictions for individuals (i.e., all regression coefficients, and model intercept or baseline survival at a given time point). | NA |
| | 15b | D | Explain how to the use the prediction model. | NA |
| Model performance | 16 | D;V | Report performance measures (with CIs) for the prediction model. | NA |
| Model-updating | 17 | V | If done, report the results from any model updating (i.e., model specification, model performance). | NA |
| **Discussion** | | | | |
| Limitations | 18 | D;V | Discuss any limitations of the study (such as nonrepresentative sample, few events per predictor, missing data). | 8 |
| Interpretation | 19a | V | For validation, discuss the results with reference to performance in the development data, and any other validation data. | NA |
| | 19b | D;V | Give an overall interpretation of the results, considering objectives, limitations, results from similar studies, and other relevant evidence. | NA |
| Implications | 20 | D;V | Discuss the potential clinical use of the model and implications for future research. | 8 |
| **Other information** | | | | |
| Supplementary information | 21 | D;V | Provide information about the availability of supplementary resources, such as study protocol, Web calculator, and data sets. | NA |
| Funding | 22 | D;V | Give the source of funding and the role of the funders for the present study. | 10 |

*Items relevant only to the development of a prediction model are denoted by D, items relating solely to a validation of a prediction model are denoted by V, and items relating to both are denoted D;V. We recommend using the TRIPOD Checklist in conjunction with the TRIPOD Explanation and Elaboration document.

For peer review only - http://bmjopen.bmj.com/site/about/guidelines.xhtml

BMJ Open

# Development and validation of a life expectancy estimator for multimorbid older adults: a cohort study protocol



| | |
|---|---|
| Journal: | *BMJ Open* |
| Manuscript ID | bmjopen-2020-048168.R1 |
| Article Type: | Protocol |
| Date Submitted by the Author: | 12-May-2021 |





| | |
|---|---|
| Complete List of Authors: | Gastens, Viktoria; University of Bern, Institute of Primary Health Care (BIHAM); University of Bern, Graduate School for Health Sciences<br>Del Giovane, Cinzia; University of Bern, Institute of Primary Health Care (BIHAM); University of Fribourg, Population Health Laboratory (#PopHealthLab)<br>Anker, Daniela; University of Bern, Institute of Primary Health Care (BIHAM); University of Fribourg, Population Health Laboratory (#PopHealthLab),<br>Feller, Martin; University of Bern, Institute of Primary Health Care (BIHAM); University of Bern, Department of General Internal Medicine, Inselspital, Bern University Hospital<br>Syrogiannouli, Lamprini; University of Bern, Institute of Primary Health Care (BIHAM)<br>Schwab, Nathalie; University of Bern, Department of General Internal Medicine, Inselspital, Bern University Hospital<br>Bauer, Douglas; University of California San Francisco, Departments of Medicine and Epidemiology & Biostatistics<br>Rodondi, Nicolas; University of Bern, Institute of Primary Health Care (BIHAM); University of Bern, Department of General Internal Medicine, Inselspital, Bern University Hospital<br>Chiolero, Arnaud; University of Fribourg, Population Health Laboratory (#PopHealthLab); McGill University, School of Global and Population Health |





| | |
|---|---|
| <b>Primary Subject Heading</b>: | Geriatric medicine |
| Secondary Subject Heading: | Epidemiology |
| Keywords: | PREVENTIVE MEDICINE, PUBLIC HEALTH, STATISTICS & RESEARCH METHODS |
| | |



SCHOLARONE™
Manuscripts

*I, the Submitting Author has the right to grant and does grant on behalf of all authors of the Work (as defined in the below author licence), an exclusive licence and/or a non-exclusive licence for contributions from authors who are: i) UK Crown employees; ii) where BMJ has agreed a CC-BY licence shall apply, and/or iii) in accordance with the terms applicable for US Federal Government officers or employees acting as part of their official duties; on a worldwide, perpetual, irrevocable, royalty-free basis to BMJ Publishing Group Ltd ("BMJ") its licensees and where the relevant Journal is co-owned by BMJ to the co-owners of the Journal, to publish the Work in this journal and any other BMJ products and to exploit all rights, as set out in our licence.*

*The Submitting Author accepts and understands that any supply made under these terms is made by BMJ to the Submitting Author unless you are acting as an employee on behalf of your employer or a postgraduate student of an affiliated institution which is paying any applicable article publishing charge ("APC") for Open Access articles. Where the Submitting Author wishes to make the Work available on an Open Access basis (and intends to pay the relevant APC), the terms of reuse of such Open Access shall be governed by a Creative Commons licence – details of these licences and which Creative Commons licence will apply to this Work are set out in our licence referred to above.*

*Other than as permitted in any relevant BMJ Author's Self Archiving Policies, I confirm this Work has not been accepted for publication elsewhere, is not being considered for publication elsewhere and does not duplicate material already published. I confirm all authors consent to publication of this Work and authorise the granting of this licence.*

For peer review only

# Development and validation of a life expectancy estimator for multimorbid older adults: a cohort study protocol

Viktoria Gastens[1,2], Cinzia Del Giovane[1,5], Daniela Anker[1,5], Martin Feller[1,3], Lamprini Syrogiannouli[1], Nathalie Schwab[1,3], Douglas C. Bauer[6], Nicolas Rodondi[1,3], and Arnaud Chiolero[1,4,5]

[1]Institute of Primary Health Care (BIHAM), University of Bern, Bern, Switzerland
[2]Graduate School for Health Sciences, University of Bern, Bern, Switzerland
[3]Department of General Internal Medicine, Inselspital, Bern University Hospital, University of Bern, Bern, Switzerland
[4]School of Global and Population Health, McGill University, Montreal, Canada
[5]Population Health Laboratory (#PopHealthLab), Department of Community Health, University of Fribourg, Fribourg, Switzerland
[6]Departments of Medicine and Epidemiology & Biostatistics, University of California, San Francisco, CA, USA

April 16, 2021

**Correspondence to**
Viktoria Gastens
Institute of Primary Health Care (BIHAM)
University of Bern
Mittelstrasse 43
3012 Bern, Switzerland
> viktoria.gastens@biham.unibe.ch

**Word count**
2040 / 4000



For peer review only - http://bmjopen.bmj.com/site/about/guidelines.xhtml

For peer review only

## ABSTRACT

### Background

Older multimorbid adults have a high risk of mortality and a short life expectancy (LE). Providing high-value care and avoiding care overuse, including of preventive care, is a serious challenge among multimorbid patients. While guidelines recommend to tailor preventive care according to the estimated LE, a LE estimator does not exist for this specific population. Our objective is therefore to develop a LE estimator for older multimorbid adults by transforming a mortality prognostic index, which will be developed and internally validated in a prospective cohort.

### Methods and analysis

We will analyse data of the OPERAM cohort study in Bern, Switzerland. 822 participants were included at hospitalisation with age of 70 years or older, multimorbidity (3 or more chronic medical conditions) and polypharmacy (use of 5 drugs or more for >30 days). All-cause mortality will be assessed during 3 years of follow-up. We will apply a flexible parametric survival model with backward stepwise selection to identify the mortality risk predictors. The model will be internally validated using bootstrapping techniques. We will derive a point-based risk score from the regression coefficients. We will transform the 3-year mortality prognostic index into a LE estimator using the Gompertz survival function. We will perform a qualitative assessment of the clinical usability of the LE estimator and its application. We will conduct the development and validation of the mortality prognostic index following the Prognosis Research Strategy (PROGRESS) framework and report it following the Transparent Reporting of a multivariable prediction model for Individual Prognosis Or Diagnosis (TRIPOD) statement.

### Ethics and dissemination

Written informed consent by patients themselves or, in the case of cognitive impairment by a legal representative, was required before enrolment. The local ethics committee (Kantonale Ethikkommission Bern) has approved the study. We plan to publish the results in peer-reviewed journals and present them at national and international conferences.


## STRENGTHS AND LIMITATIONS OF THIS STUDY

- We will provide the first life expectancy estimator specifically for older multimorbid adults.
- We use high-quality data from a large prospective cohort study of multimorbid older adults.
- This predictive tool may help clinicians to personalize preventive care according to individual life expectancy.
- External validation of the new life expectancy estimator in another cohort will still be required.

For peer review only

## BACKGROUND

Multimorbidity is frequent among older adults and is associated with a shorter life expectancy (LE), [1] a low quality of life, and a high health care utilisation such as polypharmacy [2] and multiple hospital admissions. [3] While the benefits and harms of preventive care, such as cancer screening and cardiovascular disease (CVD) preventive treatment, are well known up to the age of 75 years, they are not directly applicable to older multimorbid patients. [4, 5] For this reason, international guidelines such as USPSTF (U.S. Preventive Services Task Force) recommend accounting for LE when deciding upon preventive care.

Accounting for LE is indeed key because most preventive care has a lagtime to benefit. The lagtime to benefit is the time interval between an intervention (e.g., cancer screening) and the moment when health outcomes are improved (e.g., lower cancer mortality). It varies greatly, ranging from 6 months for statin treatment in the secondary prevention of CVD to up to 10 years for several cancer screenings. [6, 7] If the lagtime to benefit of the intervention is longer than the estimated LE, the patient cannot benefit from it [8, 9] and the intervention should not be recommended. Older multimorbid adults are at high risk of not having the time to benefit, and harms may outweigh the benefits of an intervention. To decide which preventive care should be delivered, the lagtime to benefit should be therefore weighted against the estimated LE, as proposed for cancer screening by Lee et al. [6, 7]

There is however no tool to accurately estimate LE among multimorbid patients. LE estimators are usually derived from mortality risk scores. Several risk scores allow estimating mortality risk for older adults, [8] but they are not recommended for widespread use and do not directly translate to LE estimates. To our knowledge, only Lee et al. have developed an LE estimator from a mortality risk prognostic index from a cohort of community dwelling older adults with a relatively low mortality rate, [9] but it is not directly applicable to multimorbid patients and other populations with relatively high mortality rates. Our objective is therefore to develop and internally validate a LE estimator for older multimorbid adults.



For peer review only - http://bmjopen.bmj.com/site/about/guidelines.xhtml

# METHODS AND ANALYSIS

## Source of data and study design

We will use data from 822 participants of an ongoing study in Bern, Switzerland. Participants were originally enrolled in a clinical trial with an intervention lasting 1 year and will be followed-up post-intervention for an additional two years as a cohort. The clinical trial was the cluster randomized controlled trial OPtimising thERapy to prevent Avoidable hospital admissions in Multimorbid older people (OPERAM; [10]). Participants were assigned to receive either standard care or a medication review by a Systematic Tool to Reduce Inappropriate Prescribing (STRIP) with observation of the primary outcome of drug-related hospital admission (DRA) over 1 year. For our study, we will use data collected at baseline (December 2016-October 2018) and up to 3 years after baseline (until October 2021). **Figure 1** shows the schedule of data collection for this project.

Study nurses collected baseline data by a personal interview with the participant and from medical files. Follow-ups are conducted via phone calls. Phone interviews are held with participants or relatives, otherwise with a proxy or with the general practitioner, when the participants are not reachable or not able to answer.

## Participants

822 participants were enrolled at hospitalisation in the Inselspital, University Hospital, Bern, Switzerland. Inclusion criteria were age of 70 years or older, multimorbidity (3 or more chronic medical conditions) and polypharmacy (use of 5 drugs or more for >30 days). A detailed account of the study participants' baseline characteristics is given in **Table 1**.

**Table 1.** Baseline characteristics of participants. IQR: interquartile range; BMI: body mass index; CVD: cardiovascular disease.

| Baseline characteristic | n (%) |
|---|---|
| N | 822 |
| Female | 347 (42.2) |
| Age, median [IQR] | 79 [74.0; 84.0] |
| BMI, median [IQR] | 26.0 [23.0; 29.1] |
| Smoking | 69 (8.4) |
| Education | |
| University | 151 (18.4) |
| High school | 491 (59.7) |
| Less than high school | 170 (20.7) |
| Dementia | 82 (10.0) |
| History of Cancer | 324 (39.4) |
| History of CVD | 528 (64.2) |
| CVD risk factors and medication | |
| Hypertension | 626 (76.2) |
| Hypertension with treatment | 529 (64.4) |
| Hypercholesterolemia | 309 (37.6) |
| Hypercholesterolemia with treatment | 229 (27.9) |
| Diabetes | 256 (31.1) |
| Diabetes with treatment | 202 (24.6) |



For peer review only - http://bmjopen.bmj.com/site/about/guidelines.xhtml

| | |
|---|---|
| Chronic medication, median [IQR] | 10 [6.5; 13.5] |
| Diagnoses, median [IQR] | 16 [12.5; 19.5] |
| Hospitalisations during the last year, median [IQR] | 1 [0.5; 1.5] |

Written informed consent by patients themselves or, in the case of cognitive impairment by a legal representative, had already been obtained before enrolment. Patients planned for direct admission to palliative care (<24 hours after admission), or patients undergoing a structured drug review other than the trial intervention, or who had passed a structured drug review within the last 2 months were excluded. Also patients for whom it was not possible to obtain an informed consent were excluded.

**Outcome**

The outcome of interest is all-cause mortality over the 3 years of follow-up. 153 deaths (18.6%) occurred over the first year of follow-up. Since participants were recruited during hospital admission, a higher mortality rate is expected during the first year of follow-up compared with subsequent follow-up and we expect approximately 250 deaths (30%) over the 3 years of follow-up.

**Development, validation, and clinical usability of a life expectancy estimator for multimorbid adults**

We will develop and validate the mortality prognostic index following the Prognosis Research Strategy (PROGRESS) framework, [11] and report it following the Transparent Reporting of a multivariable prediction model for Individual Prognosis Or Diagnosis (TRIPOD) statement. [12] We will further follow the recommendations of Moons et al. [13, 14] for risk prediction models and apply the method described below for 3-year mortality prediction models. **Figure 2** summarizes the process of developing the life expectancy estimator tool.

*Candidate predictors identification*

Candidate predictors are usually derived from previous research efforts in this field, [8, 15] ease and reliability of measurement in clinical setting, and background knowledge on potential associations with the outcome. Therefore, we will perform a literature review of potential predictors, and also consider factors included in the OPERAM dataset that may not be identified from the review but specific to the target population. **Table 2** shows a list of potential candidate predictors. They include demographic (age, sex), clinical characteristics (comorbidity, medication, body mass index), smoking, functional status variables, and hospitalization. Notably, we will account for the severity of comorbidity and functional impairment, which has not been considered by Lee et al. [9]

**Table 2.** Potential candidate predictors from information obtained at baseline.

| Variable | Description |
|---|---|
| Age | |
| Sex | |
| Comorbidities | Charlson Comorbidity Index (CCI) |
| Medication burden | Number of drugs before index hospitalization |
| Body-Mass-Index (BMI) | Ratio of weight (kg) / square of height ($m^2$) |



For peer review only - http://bmjopen.bmj.com/site/about/guidelines.xhtml

| | |
|---|---|
| Weight loss | Lost weight during last year (yes/no) |
| Smoking | Current smoker (yes/no) |
| Hospitalisation | Number of hospitalisations during last year |
| Activities of Daily Living (ADL) | Barthel-Index: bathing, bladder control, bowel control, dressing, feeding, grooming, mobility, stairs, toilet use, transfer |
| Generic health status | EQ-5D score: mobility, self-care, usual activities, pain/discomfort and anxiety/depression |
| Falls | Number of falls during last year |
| Nursing | Nursing home residency (yes/no) |

As part of the OPERAM trial, the participants have been assigned to a control or intervention group at baseline. We will test the predictive effect of this assignment on mortality and account for it in the analyses if necessary.

*Mortality risk prognostic model*

The relationship between candidate predictors and outcome will be analysed using the flexible parametric survival model. We will use the backward selection method. [13, 14] The remaining variables will form the final model. We will use multiple imputation for missing values under a missing at random assumption in order to reduce bias and avoid excluding participants from the analysis. [13] We will investigate the predictive accuracy of the final model by testing calibration and discrimination. We will assess the model's calibration by graphical calibration plots and by comparing the predicted to the observed mortality, with a relative difference of less than 10% considered satisfactory. [8] The model's discrimination will be assessed with Harrell's C statistic. [8] We will evaluate potential overfitting and optimism by internal validation with bootstrapping techniques. We will perform 500 bootstrap cycles in the original sample, resampling the same number of patients. In each bootstrap sample, we will derive a prediction model. We will calculate optimism as difference in performance measures between the original sample and the respective bootstrap sample. This will be repeated for all bootstrap samples to estimate the average optimism. [13, 14] If necessary, we will adjust the model for overfitting.

We have calculated the required sample size for conducting our multivariable prediction model utilizing the criteria proposed by Riley et al. [16]and implemented in the *pmsampsize* library for the R environment. [17] For the 3-year mortality risk prognostic model, the minimum sample size required with 12 candidate predictor parameters, an expected outcome event rate of 0.1 per year, and an anticipated Cox-Snell $R^2$ 0.126 (C statistic of [15] 0.82 [15, 18]) is 799 with 20 events per predictor parameter. This is considerably more than the idiomatic 10 events per predictor parameter. Our sample size of 822 is therefore adequate for this project.

To have a better clinical sense of the statistical power of our study, we estimated the power to detect a between-group difference in LE that can be considered as important on a clinical point of view. If we stratify 3 groups of equal number of patients (N=274) with a relatively short, average, and long LE, the power to detect a statistically significant between-group difference of LE of 2 to 5 years is indicated in Table 3:

**Table 3**. Power to detect a statistically significant between-group difference in LE along various assumptions (N=274 per group; alpha = 0.05; two-sided). SD: standard deviation



For peer review only - http://bmjopen.bmj.com/site/about/guidelines.xhtml

| Difference in LE (years) | SD of LE (years) | Power |
|---|---|---|
| 2 | 4 | >99% |
| 2 | 8 | 83% |
| 5 | 4 | >99% |
| 5 | 8 | >99% |

The relatively high power in all scenarios indicates that the sample size is adequate to address the research question.

*Point-based risk score*

From the final model, we will derive a point-based risk score by assigning points to each risk factor. Each $\beta$ coefficient will be divided by the lowest $\beta$ coefficient and rounded to the nearest integer. The sum of points for each risk factor will then represent the total risk score of this participant. [15]

*Life expectancy estimator*

We will transform the 3-year prognostic index into a LE estimator following the method of Lee et al. [9] In particular, we will use the new 3-year mortality prognostic index to define subpopulations with the same risk score. [9] We will fit a Gompertz survival function with each point score as a categorical predictor having a flexible proportional effect on the hazard rate. The Gompertz function assumes that each subpopulation will experience an exponential rise in mortality risk over time ($h_i(t) = \lambda_i \exp(\gamma t)$ , where $\lambda_i = \exp(x_i\beta)$). The Gompertz model will allow us to determine the time to 25%, 50% and 75% mortality for each of the risk point groups. We will report 95% confidence for the median survival intervals using bootstrapping techniques as well as 50% prediction intervals. We will compare our fit Gompertz model with observed Kaplan-Meier (KM) survival curves. The Lee LE estimator [9]will be recalibrated using standard methods to reflect the higher mortality rates of the target population. [14] The performance of the new LE estimator will be compared to the recalibrated Lee LE estimator by the Brier score [19] and Harrell's C statistic. We will build an interactive web application of the final model using the Shiny package in the R environment.

*Clinical usability of the new LE estimator*

We will perform a qualitative assessment of the LE estimator with one-to-one interviews with at least 10 clinicians. [20] We will assess whether the prediction parameters are pertinent and measurable in clinical practice, and evaluate barriers and enablers for impactful implementation of the tool. If no consent on the parameters to be included is found, we will use an iterative process, based on the Delphi-method. [21] Identified barriers and enablers will be used for the elaboration of implementation strategies.

## ETHICS AND DISSEMINATION

The local ethics committee (Kantonale Ethikkommission Bern) has approved the study protocol. All participants and their data are handled according to the ethical principles of the Declaration of Helsinki. This study complies with all applicable standards of the International Conference on Harmonisation E6 Guideline for Good Clinical Practice (ICH-GCP 1996) guideline. We plan to publish the results from our study in peer-reviewed and open access journals and present them at national and international conferences. Data will be deposited in the Bern Open Repository and Information System (BORIS) (www.boris.unibe.ch).



For peer review only - http://bmjopen.bmj.com/site/about/guidelines.xhtml

**PERSPECTIVE**

To our knowledge, we will be the first to develop an LE estimator specifically for older multimorbid adults. A major strength of this study is its high feasibility due to the follow-up of 822 already included multimorbid participants with strong strategies of containment of loss to follow-up. Notably, individuals with cognitive impairment were included, who are commonly excluded from studies. In addition to the development and internal validation of the LE estimator, we also assess its implementation and clinical usability.

One major limitation is the lack of external validation but we will explore opportunities to test the LE estimator in a different dataset of older multimorbid adults. As the study participants were included at the time of hospitalization, they may not be representative of all patients with multimorbidity, and this could reduce the transportability of our findings to other populations. Further, we have a relatively short duration of follow-up.

Our results will be useful for both clinical and research activities as they can have a major impact on preventive care practice by helping health care providers to tailor preventive care according to the estimated LE. Eventually, our study will help preventing under- and overuse of preventive care in the growing older population.

For peer review only



For peer review only - http://bmjopen.bmj.com/site/about/guidelines.xhtml

# References

[1] A. Menotti, I. Mulder, A. Nissinen, S. Giampaoli, E. J. M. Feskens and D. Kromhout, "Prevalence of morbidity and multimorbidity in elderly male populations and their impact on 10-year all-cause mortality: The FINE study (Finland, Italy, Netherlands, Elderly)," *Journal of clinical epidemiology,* vol. 54, p. 680–686, 2001.

[2] C. E. Aubert, S. Streit, B. R. Da Costa, T.-H. Collet, J. Cornuz, J.-M. Gaspoz, D. Bauer, D. Aujesky and N. Rodondi, "Polypharmacy and specific comorbidities in university primary care settings," *European journal of internal medicine,* vol. 35, p. 35–42, 2016.

[3] A. Cassell, D. Edwards, A. Harshfield, K. Rhodes, J. Brimicombe, R. Payne and S. Griffin, "The epidemiology of multimorbidity in primary care: a retrospective cohort study," *Br J Gen Pract,* vol. 68, p. e245–e251, 2018.

[4] J. B. Schwartz, "Primary prevention: Do the very elderly require a different approach?," *Trends in Cardiovascular Medicine,* vol. 25, pp. 228-239, 2015.

[5] I. Lansdorp-Vogelaar, R. Gulati, A. B. Mariotto, C. B. Schechter, T. M. De Carvalho, A. B. Knudsen, N. T. Van Ravesteyn, E. A. M. Heijnsdijk, C. Pabiniak, M. Van Ballegooijen and others, "Personalizing age of cancer screening cessation based on comorbid conditions: model estimates of harms and benefits," *Annals of internal medicine,* vol. 161, p. 104–112, 2014.

[6] S. J. Lee, W. J. Boscardin, I. Stijacic-Cenzer, J. Conell-Price, S. O\textquoterightBrien and L. C. Walter, "Time lag to benefit after screening for breast and colorectal cancer: meta-analysis of survival data from the United States, Sweden, United Kingdom, and Denmark," *BMJ,* vol. 346, 2013.

[7] S. J. Lee, R. M. Leipzig and L. C. Walter, "Incorporating Lag Time to Benefit Into Prevention Decisions for Older Adults," *JAMA,* vol. 310, pp. 2609-2610, 12 2013.

[8] L. C. Yourman, S. J. Lee, M. A. Schonberg, E. W. Widera and A. K. Smith, "Prognostic Indices for Older Adults: A Systematic Review," *JAMA,* vol. 307, pp. 182-192, 1 2012.

[9] S. J. Lee, W. J. Boscardin, K. A. Kirby and K. E. Covinsky, "Individualizing Life Expectancy Estimates for Older Adults Using the Gompertz Law of Human Mortality," *PLOS ONE,* vol. 9, pp. 1-8, 9 2014.

[10] L. Adam, E. Moutzouri, C. Baumgartner, A. L. Loewe, M. Feller, K. M\textquoterightRabet-Bensalah, N. Schwab, S. Hossmann, C. Schneider, S. Jegerlehner, C. Floriani, A. Limacher, K. T. Jungo, C. J. A. Huibers, S. Streit, M. Schwenkglenks, M. Spruit, A. Van Dorland, J. Donzé, P. M. Kearney, P. Jüni, D. Aujesky, P. Jansen, B. Boland, O. Dalleur, S. Byrne, W. Knol, A. Spinewine, D. O\textquoterightMahony, S. Trelle and N. Rodondi, "Rationale and design of OPtimising thERapy to prevent Avoidable hospital admissions in Multimorbid older people (OPERAM): a cluster randomised controlled trial," *BMJ Open,* vol. 9, 2019.

[11] E. W. Steyerberg, K. G. M. Moons, D. A. van der Windt, J. A. Hayden, P. Perel, S. Schroter, R. D. Riley, H. Hemingway, D. G. Altman, P. R. O. G. R. E. S. S. Group and others, "Prognosis Research Strategy (PROGRESS) 3: prognostic model research," *PLoS medicine,* vol. 10, 2013.

[12] G. S. Collins, J. B. Reitsma, D. G. Altman and K. G. M. Moons, "Transparent reporting of a multivariable prediction model for individual prognosis or diagnosis (TRIPOD): the TRIPOD statement," *BMJ,* vol. 350, 2015.

[13] K. G. M. Moons, A. P. Kengne, M. Woodward, P. Royston, Y. Vergouwe, D. G. Altman and D. E. Grobbee, "Risk prediction models: I. Development, internal validation, and assessing the incremental value of a new (bio)marker," *Heart,* vol. 98, p. 683–690, 2012.

[14] K. G. M. Moons, A. P. Kengne, D. E. Grobbee, P. Royston, Y. Vergouwe, D. G. Altman and M. Woodward, "Risk prediction models: II. External validation, model updating, and impact assessment," *Heart,* vol. 98, p. 691–698, 2012.

[15] S. J. Lee, K. Lindquist, M. R. Segal and K. E. Covinsky, "Development and Validation of a Prognostic



For peer review only - http://bmjopen.bmj.com/site/about/guidelines.xhtml

Index for 4-Year Mortality in Older Adults," *JAMA,* vol. 295, pp. 801-808, 2 2006.
[16] R. D. Riley, J. Ensor, K. I. E. Snell, F. E. Harrell, G. P. Martin, J. B. Reitsma, K. G. M. Moons, G. Collins and M. van Smeden, "Calculating the sample size required for developing a clinical prediction model," *BMJ,* vol. 368, 2020.
[17] J. Ensor, E. C. Martin and R. D. Riley, "pmsampsize: Calculates the Minimum Sample Size Required for Developing a Multivariable Prediction Model," 2019.
[18] R. D. Riley, K. I. E. Snell, J. Ensor, D. L. Burke, F. E. Harrell Jr, K. G. M. Moons and G. S. Collins, "Minimum sample size for developing a multivariable prediction model: PART II - binary and time-to-event outcomes," *Statistics in Medicine,* vol. 38, pp. 1276-1296, 2019.
[19] N. R. Cook, "Use and Misuse of the Receiver Operating Characteristic Curve in Risk Prediction," *Circulation,* vol. 115, pp. 928-935, 2007.
[20] R. L. Breen, "A practical guide to focus-group research," *Journal of Geography in Higher Education,* vol. 30, p. 463–475, 2006.
[21] R. A. N. D. Corporation, "Delphi Method," [Online]. Available: https://www.rand.org/topics/delphi-method.html.

## Authors' contributions

Study concept and design: AC, NR, CDG. Acquisition, analysis, interpretation of data: NS, VG, CDG, LS. Drafting of the article: VG, CDG, AC. Critical revision of the article: VG, CDG, DA, MF, LS, NS, DB, NR, AC. Statistical analysis: VG, LS, CDG. Obtained funding: AC. Administrative, technical or material support: NS. Supervision: AC, CDG.

## Funding

This work was supported by the Swiss National Science Foundation grant number (320030_188549/01 to Prof Chiolero). This work is part of the project "OPERAM: OPtimising thERapy to prevent Avoidable hospital admissions in the Multimorbid elderly" supported by the European Union's Horizon 2020 research and innovation programme under the grant agreement No 6342388, and by the Swiss State Secretariat for Education, Research and Innovation (SERI) under contract number 15.0137. The opinions expressed and arguments employed herein are those of the authors and do not necessarily reflect the official views of the EC and the Swiss government.

## Competing interests

None declared.

## Ethics approval

The local Ethics Committee in Bern, Switzerland, approved the study protocol with the project number 2018-00784.



For peer review only

For peer review only - http://bmjopen.bmj.com/site/about/guidelines.xhtml

**Figure legends**

Figure 1: Schedule of data collection from baseline to 3-year follow-up.

Figure 2: Flowchart of the development steps (boxes) and methods used (arrows) for the life expectancy estimator tool.

For peer review only



For peer review only - http://bmjopen.bmj.com/site/about/guidelines.xhtml

Inclusion:
- ≥70 years
- Multimorbidity (≥3 conditions)
- Polypharmacy (≥5 drugs)

Exclusion:
- Admission to palliative care (<24 h)
- Structured drug review (<2 months)
- No informed consent

| | **Baseline** First patient first visit 12/2016 | **1 year follow-up** | **2 year follow-up** | **3 year follow-up** Last patient last visit 10/2021 |
|---|---|---|---|---|
| Baseline characteristics | X | | | |
| Activities of Daily Living (ADL) | X | X | | |
| Quality of Life (EQ-5D) | X | X | | |
| Falls and fractures | X | X | | |
| Health economics | X | X | | |
| Diagnoses | X | X | X | X |
| Medication | X | X | X | X |
| Blood pressure and cholesterol | X | X | X | X |
| Life expectancy questionnaire by Lee et al.[1] | | X | X | X |
| Cancer screening | | X | X | X |

[1] SJ Lee et al. "Development and Validation of a Prognostic Index for 4-Year Mortality in Older Adults". *JAMA* 2006

Schedule of data collection from baseline to 3-year follow-up.

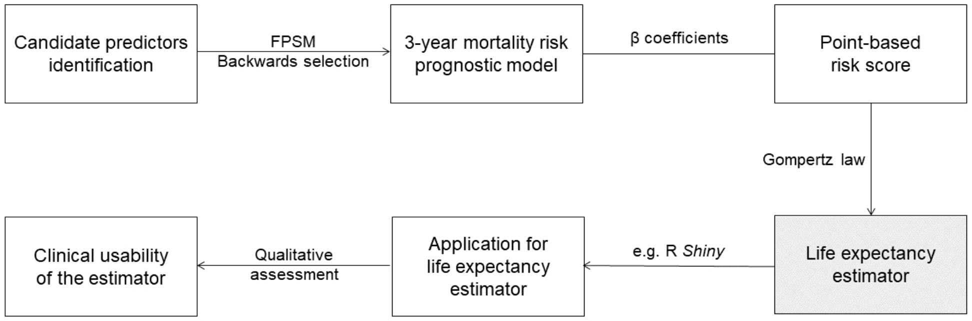


Flowchart of the development steps (boxes) and methods used (arrows) for the life expectancy estimator tool.

# TRIPOD Checklist: Prediction Model Development and Validation

| Section/Topic | Item | | Checklist Item | Page |
|---|---|---|---|---|
| **Title and abstract** | | | | |
| Title | 1 | D;V | Identify the study as developing and/or validating a multivariable prediction model, the target population, and the outcome to be predicted. | 1 |
| Abstract | 2 | D;V | Provide a summary of objectives, study design, setting, participants, sample size, predictors, outcome, statistical analysis, results, and conclusions. | 2 |
| **Introduction** | | | | |
| Background and objectives | 3a | D;V | Explain the medical context (including whether diagnostic or prognostic) and rationale for developing or validating the multivariable prediction model, including references to existing models. | 3 |
| | 3b | D;V | Specify the objectives, including whether the study describes the development or validation of the model or both. | 3 |
| **Methods** | | | | |
| Source of data | 4a | D;V | Describe the study design or source of data (e.g., randomized trial, cohort, or registry data), separately for the development and validation data sets, if applicable. | 4 |
| | 4b | D;V | Specify the key study dates, including start of accrual; end of accrual; and, if applicable, end of follow-up. | 4 |
| Participants | 5a | D;V | Specify key elements of the study setting (e.g., primary care, secondary care, general population) including number and location of centres. | 4 |
| | 5b | D;V | Describe eligibility criteria for participants. | 4 |
| | 5c | D;V | Give details of treatments received, if relevant. | 4 |
| Outcome | 6a | D;V | Clearly define the outcome that is predicted by the prediction model, including how and when assessed. | 5 |
| | 6b | D;V | Report any actions to blind assessment of the outcome to be predicted. | NA |
| Predictors | 7a | D;V | Clearly define all predictors used in developing or validating the multivariable prediction model, including how and when they were measured. | 6 |
| | 7b | D;V | Report any actions to blind assessment of predictors for the outcome and other predictors. | NA |
| Sample size | 8 | D;V | Explain how the study size was arrived at. | 7 |
| Missing data | 9 | D;V | Describe how missing data were handled (e.g., complete-case analysis, single imputation, multiple imputation) with details of any imputation method. | 7 |
| Statistical analysis methods | 10a | D | Describe how predictors were handled in the analyses. | 7 |
| | 10b | D | Specify type of model, all model-building procedures (including any predictor selection), and method for internal validation. | 7 |
| | 10c | V | For validation, describe how the predictions were calculated. | 7 |
| | 10d | D;V | Specify all measures used to assess model performance and, if relevant, to compare multiple models. | 7 |
| | 10e | V | Describe any model updating (e.g., recalibration) arising from the validation, if done. | 7 |
| Risk groups | 11 | D;V | Provide details on how risk groups were created, if done. | 7 |
| Development vs. validation | 12 | V | For validation, identify any differences from the development data in setting, eligibility criteria, outcome, and predictors. | NA |
| **Results** | | | | |
| Participants | 13a | D;V | Describe the flow of participants through the study, including the number of participants with and without the outcome and, if applicable, a summary of the follow-up time. A diagram may be helpful. | NA |
| | 13b | D;V | Describe the characteristics of the participants (basic demographics, clinical features, available predictors), including the number of participants with missing data for predictors and outcome. | NA |
| | 13c | V | For validation, show a comparison with the development data of the distribution of important variables (demographics, predictors and outcome). | NA |
| Model development | 14a | D | Specify the number of participants and outcome events in each analysis. | NA |
| | 14b | D | If done, report the unadjusted association between each candidate predictor and outcome. | NA |
| Model specification | 15a | D | Present the full prediction model to allow predictions for individuals (i.e., all regression coefficients, and model intercept or baseline survival at a given time point). | NA |
| | 15b | D | Explain how to the use the prediction model. | NA |
| Model performance | 16 | D;V | Report performance measures (with CIs) for the prediction model. | NA |
| Model-updating | 17 | V | If done, report the results from any model updating (i.e., model specification, model performance). | NA |
| **Discussion** | | | | |
| Limitations | 18 | D;V | Discuss any limitations of the study (such as nonrepresentative sample, few events per predictor, missing data). | 8 |
| Interpretation | 19a | V | For validation, discuss the results with reference to performance in the development data, and any other validation data. | NA |
| | 19b | D;V | Give an overall interpretation of the results, considering objectives, limitations, results from similar studies, and other relevant evidence. | NA |
| Implications | 20 | D;V | Discuss the potential clinical use of the model and implications for future research. | 8 |
| **Other information** | | | | |
| Supplementary information | 21 | D;V | Provide information about the availability of supplementary resources, such as study protocol, Web calculator, and data sets. | NA |
| Funding | 22 | D;V | Give the source of funding and the role of the funders for the present study. | 10 |

*Items relevant only to the development of a prediction model are denoted by D, items relating solely to a validation of a prediction model are denoted by V, and items relating to both are denoted D;V. We recommend using the TRIPOD Checklist in conjunction with the TRIPOD Explanation and Elaboration document.

For peer review only - http://bmjopen.bmj.com/site/about/guidelines.xhtml

BMJ Open

# Development and validation of a life expectancy estimator for multimorbid older adults: a cohort study protocol

| | |
|---|---|
| Journal: | *BMJ Open* |
| Manuscript ID | bmjopen-2020-048168.R2 |
| Article Type: | Protocol |
| Date Submitted by the Author: | 19-Jul-2021 |
| Complete List of Authors: | Gastens, Viktoria; University of Bern, Institute of Primary Health Care (BIHAM); University of Bern, Graduate School for Health Sciences<br>Del Giovane, Cinzia; University of Bern, Institute of Primary Health Care (BIHAM); University of Fribourg, Population Health Laboratory (#PopHealthLab)<br>Anker, Daniela; University of Bern, Institute of Primary Health Care (BIHAM); University of Fribourg, Population Health Laboratory (#PopHealthLab),<br>Feller, Martin; University of Bern, Institute of Primary Health Care (BIHAM); University of Bern, Department of General Internal Medicine, Inselspital, Bern University Hospital<br>Syrogiannouli, Lamprini; University of Bern, Institute of Primary Health Care (BIHAM)<br>Schwab, Nathalie; University of Bern, Department of General Internal Medicine, Inselspital, Bern University Hospital<br>Bauer, Douglas; University of California San Francisco, Departments of Medicine and Epidemiology & Biostatistics<br>Rodondi, Nicolas; University of Bern, Institute of Primary Health Care (BIHAM); University of Bern, Department of General Internal Medicine, Inselspital, Bern University Hospital<br>Chiolero, Arnaud; University of Fribourg, Population Health Laboratory (#PopHealthLab); McGill University, School of Global and Population Health |
| <b>Primary Subject Heading</b>: | Epidemiology |
| Secondary Subject Heading: | Geriatric medicine |
| Keywords: | PREVENTIVE MEDICINE, PUBLIC HEALTH, STATISTICS & RESEARCH METHODS |
| | |

SCHOLARONE™
Manuscripts

*I, the Submitting Author has the right to grant and does grant on behalf of all authors of the Work (as defined in the below author licence), an exclusive licence and/or a non-exclusive licence for contributions from authors who are: i) UK Crown employees; ii) where BMJ has agreed a CC-BY licence shall apply, and/or iii) in accordance with the terms applicable for US Federal Government officers or employees acting as part of their official duties; on a worldwide, perpetual, irrevocable, royalty-free basis to BMJ Publishing Group Ltd ("BMJ") its licensees and where the relevant Journal is co-owned by BMJ to the co-owners of the Journal, to publish the Work in this journal and any other BMJ products and to exploit all rights, as set out in our licence.*

*The Submitting Author accepts and understands that any supply made under these terms is made by BMJ to the Submitting Author unless you are acting as an employee on behalf of your employer or a postgraduate student of an affiliated institution which is paying any applicable article publishing charge ("APC") for Open Access articles. Where the Submitting Author wishes to make the Work available on an Open Access basis (and intends to pay the relevant APC), the terms of reuse of such Open Access shall be governed by a Creative Commons licence – details of these licences and which Creative Commons licence will apply to this Work are set out in our licence referred to above.*

*Other than as permitted in any relevant BMJ Author's Self Archiving Policies, I confirm this Work has not been accepted for publication elsewhere, is not being considered for publication elsewhere and does not duplicate material already published. I confirm all authors consent to publication of this Work and authorise the granting of this licence.*

For peer review only

# Development and validation of a life expectancy estimator for multimorbid older adults: a cohort study protocol

Viktoria Gastens[1,2], Cinzia Del Giovane[1,5], Daniela Anker[1,5], Martin Feller[1,3], Lamprini Syrogiannouli[1], Nathalie Schwab[1,3], Douglas C. Bauer[6], Nicolas Rodondi[1,3], and Arnaud Chiolero[1,4,5]

[1]Institute of Primary Health Care (BIHAM), University of Bern, Bern, Switzerland
[2]Graduate School for Health Sciences, University of Bern, Bern, Switzerland
[3]Department of General Internal Medicine, Inselspital, Bern University Hospital, University of Bern, Bern, Switzerland
[4]School of Global and Population Health, McGill University, Montreal, Canada
[5]Population Health Laboratory (#PopHealthLab), Department of Community Health, University of Fribourg, Fribourg, Switzerland
[6]Departments of Medicine and Epidemiology & Biostatistics, University of California, San Francisco, CA, USA

June, 2021

**Correspondence to**
Viktoria Gastens
Institute of Primary Health Care (BIHAM)
University of Bern
Mittelstrasse 43
3012 Bern, Switzerland
> viktoria.gastens@biham.unibe.ch

**Word count**
2100 / 4000

For peer review only

## ABSTRACT

### Background

Older multimorbid adults have a high risk of mortality and a short life expectancy (LE). Providing high-value care and avoiding care overuse, including of preventive care, is a serious challenge among multimorbid patients. While guidelines recommend to tailor preventive care according to the estimated LE, a LE estimator does not exist for this specific population. Our objective is therefore to develop a LE estimator for older multimorbid adults by transforming a mortality prognostic index, which will be developed and internally validated in a prospective cohort.

### Methods and analysis

We will analyse data of the OPERAM cohort study in Bern, Switzerland. 822 participants were included at hospitalisation with age of 70 years or older, multimorbidity (3 or more chronic medical conditions) and polypharmacy (use of 5 drugs or more for >30 days). All-cause mortality will be assessed during 3 years of follow-up. We will apply a flexible parametric survival model with backward stepwise selection to identify the mortality risk predictors. The model will be internally validated using bootstrapping techniques. We will derive a point-based risk score from the regression coefficients. We will transform the 3-year mortality prognostic index into a LE estimator using the Gompertz survival function. We will perform a qualitative assessment of the clinical usability of the LE estimator and its application. We will conduct the development and validation of the mortality prognostic index following the Prognosis Research Strategy (PROGRESS) framework and report it following the Transparent Reporting of a multivariable prediction model for Individual Prognosis Or Diagnosis (TRIPOD) statement.

### Ethics and dissemination

Written informed consent by patients themselves or, in the case of cognitive impairment by a legal representative, was required before enrolment. The local ethics committee (Kantonale Ethikkommission Bern) has approved the study. We plan to publish the results in peer-reviewed journals and present them at national and international conferences.


## STRENGTHS AND LIMITATIONS OF THIS STUDY

- We will provide the first life expectancy estimator specifically for older multimorbid adults.
- We use high-quality data from a large prospective cohort study of multimorbid older adults.
- This predictive tool may help clinicians to personalize preventive care according to individual life expectancy.
- External validation of the new life expectancy estimator in another cohort will still be required.



For peer review only - http://bmjopen.bmj.com/site/about/guidelines.xhtml

## BACKGROUND

Multimorbidity is frequent among older adults and is associated with a shorter life expectancy (LE), [1] a low quality of life, and a high health care utilisation such as polypharmacy [2] and multiple hospital admissions. [3] While the benefits and harms of preventive care, such as cancer screening and cardiovascular disease (CVD) preventive treatment, are well known up to the age of 75 years, they are not directly applicable to older multimorbid patients. [4, 5] For this reason, international guidelines such as USPSTF (U.S. Preventive Services Task Force) recommend accounting for LE when deciding upon preventive care.

Accounting for LE is indeed key because most preventive care has a lagtime to benefit. The lagtime to benefit is the time interval between an intervention (e.g., cancer screening) and the moment when health outcomes are improved (e.g., lower cancer mortality). It varies greatly, ranging from 6 months for statin treatment in the secondary prevention of CVD to up to 10 years for several cancer screenings. [6, 7] If the lagtime to benefit of the intervention is longer than the estimated LE, the patient cannot benefit from it [8, 9] and the intervention should not be recommended. Older multimorbid adults are at high risk of not having the time to benefit, and harms may outweigh the benefits of an intervention. To decide which preventive care should be delivered, the lagtime to benefit should be therefore weighted against the estimated LE, as proposed for cancer screening by Lee et al. [6, 7]

There is however no tool to accurately estimate LE among multimorbid patients. LE estimators are usually derived from mortality risk scores. Several risk scores allow estimating mortality risk for older adults, [8] but they are not recommended for widespread use and do not directly translate to LE estimates. To our knowledge, only Lee et al. have developed an LE estimator from a mortality risk prognostic index from a cohort of community dwelling older adults with a relatively low mortality rate, [9] but it is not directly applicable to multimorbid patients and other populations with relatively high mortality rates. Our objective is therefore to develop and internally validate a LE estimator for older multimorbid adults.

For peer review only



For peer review only - http://bmjopen.bmj.com/site/about/guidelines.xhtml

## METHODS AND ANALYSIS

### Source of data and study design

We will use data from 822 participants of an ongoing study in Bern, Switzerland. Participants were originally enrolled in a clinical trial with an intervention lasting 1 year and will be followed-up post-intervention for an additional two years as a cohort. The clinical trial was the cluster randomized controlled trial OPtimising thERapy to prevent Avoidable hospital admissions in Multimorbid older people (OPERAM; [10]). Participants were assigned to receive either standard care or a medication review by a Systematic Tool to Reduce Inappropriate Prescribing (STRIP) with observation of the primary outcome of drug-related hospital admission (DRA) over 1 year. For our study, we will use data collected at baseline (December 2016-October 2018) and up to 3 years after baseline (until October 2021). **Figure 1** shows the schedule of data collection for this project.

Study nurses collected baseline data by a personal interview with the participant and from medical files. Follow-ups are conducted via phone calls. Phone interviews are held with participants or relatives, otherwise with a proxy or with the general practitioner, when the participants are not reachable or not able to answer.

### Participants

822 participants were enrolled at hospitalisation in the Inselspital, University Hospital, Bern, Switzerland. Inclusion criteria were age of 70 years or older, multimorbidity (3 or more chronic medical conditions) and polypharmacy (use of 5 drugs or more for >30 days). We defined multimorbidity as presence of 3 or more coexistent chronic medical conditions with an estimated duration of 6 months or more. The medical conditions were recorded as ICD-10 codes. The estimated duration was based on a clinical decision. The ten most frequent chronic medical conditions were in descending order: essential hypertension (I10), type 2 diabetes mellitus (E11), hypertensive heart disease (I11), chronic kidney disease stage 3 (N18.3), disorders of lipoprotein metabolism and other lipidaemias (E78), atrial fibrillation and flutter (I48), chronic ischaemic heart disease (I25), hyperplasia of prostate (N40), other postsurgical states (Z98), and obesity (E66). A detailed account of the study participants' baseline characteristics is given in **Table 1**.

**Table 1.** Baseline characteristics of participants. IQR: interquartile range; BMI: body mass index; CVD: cardiovascular disease.

| Baseline characteristic | n (%) |
|---|---|
| N | 822 |
| Female | 347 (42.2) |
| Age, median [IQR] | 79 [74.0; 84.0] |
| BMI, median [IQR] | 26.0 [23.0; 29.1] |
| Smoking | 69 (8.4) |
| Education | |
| University | 151 (18.4) |
| High school | 491 (59.7) |
| Less than high school | 170 (20.7) |
| Dementia | 82 (10.0) |



For peer review only - http://bmjopen.bmj.com/site/about/guidelines.xhtml

For peer review only

| | |
|---|---|
| History of Cancer | 324 (39.4) |
| History of CVD | 528 (64.2) |
| CVD risk factors and medication | |
| Hypertension | 626 (76.2) |
| Hypertension with treatment | 529 (64.4) |
| Hypercholesterolemia | 309 (37.6) |
| Hypercholesterolemia with treatment | 229 (27.9) |
| Diabetes | 256 (31.1) |
| Diabetes with treatment | 202 (24.6) |
| Chronic medication, median [IQR] | 10 [6.5; 13.5] |
| Diagnoses, median [IQR] | 16 [12.5; 19.5] |
| Hospitalisations during the last year, median [IQR] | 1 [0.5; 1.5] |

Written informed consent by patients themselves or, in the case of cognitive impairment by a legal representative, had already been obtained before enrolment. Patients planned for direct admission to palliative care (<24 hours after admission), or patients undergoing a structured drug review other than the trial intervention, or who had passed a structured drug review within the last 2 months were excluded. Also patients for whom it was not possible to obtain an informed consent were excluded.

**Outcome**

The outcome of interest is all-cause mortality over the 3 years of follow-up. 153 deaths (18.6%) occurred over the first year of follow-up. Since participants were recruited during hospital admission, a higher mortality rate is expected during the first year of follow-up compared with subsequent follow-up and we expect approximately 250 deaths (30%) over the 3 years of follow-up.

**Development, validation, and clinical usability of a life expectancy estimator for multimorbid adults**

We will develop and validate the mortality prognostic index following the Prognosis Research Strategy (PROGRESS) framework, [11] and report it following the Transparent Reporting of a multivariable prediction model for Individual Prognosis Or Diagnosis (TRIPOD) statement. [12] We will further follow the recommendations of Moons et al. [13, 14] for risk prediction models and apply the method described below for 3-year mortality prediction models. **Figure 2** summarizes the process of developing the life expectancy estimator tool.

*Candidate predictors identification*

Candidate predictors are usually derived from previous research efforts in this field, [8, 15] ease and reliability of measurement in clinical setting, and background knowledge on potential associations with the outcome. Therefore, we will perform a literature review of potential predictors, and also consider factors included in the OPERAM dataset that may not be identified from the review but specific to the target population. **Table 2** shows a list of potential candidate predictors. They include demographic (age, sex), clinical characteristics (comorbidity, medication, body mass index), smoking, functional status variables, and hospitalization. Notably, we will account for the severity of comorbidity and functional impairment, which has not been considered



For peer review only - http://bmjopen.bmj.com/site/about/guidelines.xhtml

by Lee et al. [9]

**Table 2.** Potential candidate predictors from information obtained at baseline.

| Variable | Description |
|---|---|
| Age | |
| Sex | |
| Comorbidities | Charlson Comorbidity Index (CCI) |
| Medication burden | Number of drugs before index hospitalization |
| Body-Mass-Index (BMI) | Ratio of weight (kg) / square of height ($m^2$) |
| Weight loss | Lost weight during last year (yes/no) |
| Smoking | Current smoker (yes/no) |
| Hospitalisation | Number of hospitalisations during last year |
| Activities of Daily Living (ADL) | Barthel-Index: bathing, bladder control, bowel control, dressing, feeding, grooming, mobility, stairs, toilet use, transfer |
| Generic health status | EQ-5D score: mobility, self-care, usual activities, pain/discomfort and anxiety/depression |
| Falls | Number of falls during last year |
| Nursing | Nursing home residency (yes/no) |

As part of the OPERAM trial, the participants have been assigned to a control or intervention group at baseline. We will test the predictive effect of this assignment on mortality and account for it in the analyses if necessary.

*Mortality risk prognostic model*

The relationship between candidate predictors and outcome will be analysed using the flexible parametric survival model. We will use the backward selection method. [13, 14] The remaining variables will form the final model. We will use multiple imputation for missing values under a missing at random assumption in order to reduce bias and avoid excluding participants from the analysis. [13] We will investigate the predictive accuracy of the final model by testing calibration and discrimination. We will assess the model's calibration by graphical calibration plots and by comparing the predicted to the observed mortality, with a relative difference of less than 10% considered satisfactory. [8] The model's discrimination will be assessed with Harrell's C statistic. [8] We will use bootstrapping techniques to internally validate our mortality prognostic index. We will perform 500 bootstrap cycles from the original sample, sampling the same number of patients with replacement. In each bootstrap sample, we will derive a mortality prediction model and the relative risk score, as done in the original sample. We will also evaluate potential overfitting and optimism. We will calculate optimism as difference in performance measure (e.g. C-Statistics) between the original sample and the respective bootstrap sample. This will be repeated for all bootstrap samples to estimate the average optimism. [13, 14] If necessary, we will adjust the model for overfitting.

We have calculated the required sample size for conducting our multivariable prediction model utilizing the criteria proposed by Riley et al. [16]and implemented in the *pmsampsize* library for the R environment. [17] For the 3-year mortality risk prognostic model, the minimum sample size required with 12 candidate predictor parameters, an expected outcome event rate of 0.1 per year, and an anticipated Cox-Snell $R^2$ 0.126 (C statistic of [15] 0.82 [15, 18]) is 799 with 20 events per



For peer review only - http://bmjopen.bmj.com/site/about/guidelines.xhtml

predictor parameter. This is considerably more than the idiomatic 10 events per predictor parameter. Our sample size of 822 is therefore adequate for this project.

To have a better clinical sense of the statistical power of our study, we computed the power to detect a between-group difference in estimated LE that can be considered as important on a clinical point of view. If we stratify our sample in 3 groups of equal number of patients (N=274) with a relatively short, average, and long estimated LE, the power to detect a statistically significant between-group difference of LE of 1 to 3 years is indicated in Table 3:

**Table 3**. Power to detect a statistically significant between-group difference in LE along various assumptions (N=274 per group; alpha = 0.05; two-sided). SD: standard deviation

| Difference in LE (years) | SD of LE (years) | Power |
|---|---|---|
| 1 | 4 | 83% |
| 1 | 8 | 31% |
| 2 | 4 | >99% |
| 2 | 8 | 83% |
| 3 | 4 | >99% |
| 3 | 8 | >99% |

The relatively high power in most scenarios indicates that the sample size is adequate to address the research question.

*Point-based risk score*

From the final model, we will derive a point-based risk score by assigning points to each risk factor. Each $\beta$ coefficient will be divided by the lowest $\beta$ coefficient and rounded to the nearest integer. The sum of points for each risk factor will then represent the total risk score of this participant. [15]

*Life expectancy estimator*

We will transform the 3-year prognostic index into a LE estimator following the method of Lee et al. [9] In particular, we will use the new 3-year mortality prognostic index to define subpopulations with the same risk score. [9] We will fit a Gompertz survival function with each point score as a categorical predictor having a flexible proportional effect on the hazard rate. The Gompertz function assumes that each subpopulation will experience an exponential rise in mortality risk over time ($h_i(t) = \lambda_i \exp(\gamma t)$ , where $\lambda_i = \exp(x_i\beta)$). The Gompertz model will allow us to determine the time to 25%, 50% and 75% mortality for each of the risk point groups. We will report 95% confidence for the median survival intervals using bootstrapping techniques as well as 50% prediction intervals. We will compare our fit Gompertz model with observed Kaplan-Meier (KM) survival curves. The Lee LE estimator [9]will be recalibrated using standard methods to reflect the higher mortality rates of the target population. [14] The performance of the new LE estimator will be compared to the recalibrated Lee LE estimator by the Brier score [19] and Harrell's C statistic. We will build an interactive web application of the final model using the Shiny package in the R environment.

*Clinical usability of the new LE estimator*

We will perform a qualitative assessment of the LE estimator with one-to-one interviews with at least 10 clinicians. [20] We will assess whether the prediction parameters are pertinent and measurable in clinical practice, and evaluate barriers and enablers for impactful implementation of the tool. If no consent on the parameters to be included is found, we will use an iterative process,



For peer review only - http://bmjopen.bmj.com/site/about/guidelines.xhtml

based on the Delphi-method. [21] Identified barriers and enablers will be used for the elaboration of implementation strategies.

**Patient and Public Involvement**

Patients or the public were not involved in the design, or conduct, or reporting, or dissemination plans of our research.

## ETHICS AND DISSEMINATION

The local ethics committee (Kantonale Ethikkommission Bern) has approved the study protocol. All participants and their data are handled according to the ethical principles of the Declaration of Helsinki. This study complies with all applicable standards of the International Conference on Harmonisation E6 Guideline for Good Clinical Practice (ICH-GCP 1996) guideline. We plan to publish the results from our study in peer-reviewed and open access journals and present them at national and international conferences. Data will be deposited in the Bern Open Repository and Information System (BORIS) (www.boris.unibe.ch).

## PERSPECTIVE

To our knowledge, we will be the first to develop an LE estimator specifically for older multimorbid adults. A major strength of this study is its high feasibility due to the follow-up of 822 already included multimorbid participants with strong strategies of containment of loss to follow-up. Notably, individuals with cognitive impairment were included, who are commonly excluded from studies. In addition to the development and internal validation of the LE estimator, we also assess its implementation and clinical usability.

One major limitation is the lack of external validation but we will explore opportunities to test the LE estimator in a different dataset of older multimorbid adults. As the study participants were included at the time of hospitalization, they may not be representative of all patients with multimorbidity, and this could reduce the transportability of our findings to other populations. Further, we have a relatively short duration of follow-up.

Our results will be useful for both clinical and research activities as they can have a major impact on preventive care practice by helping health care providers to tailor preventive care according to the estimated LE. Eventually, our study will help preventing under- and overuse of preventive care in the growing older population.



For peer review only - http://bmjopen.bmj.com/site/about/guidelines.xhtml

# References

[1] A. Menotti, I. Mulder, A. Nissinen, S. Giampaoli, E. J. M. Feskens and D. Kromhout, "Prevalence of morbidity and multimorbidity in elderly male populations and their impact on 10-year all-cause mortality: The FINE study (Finland, Italy, Netherlands, Elderly)," *Journal of clinical epidemiology,* vol. 54, p. 680–686, 2001.

[2] C. E. Aubert, S. Streit, B. R. Da Costa, T.-H. Collet, J. Cornuz, J.-M. Gaspoz, D. Bauer, D. Aujesky and N. Rodondi, "Polypharmacy and specific comorbidities in university primary care settings," *European journal of internal medicine,* vol. 35, p. 35–42, 2016.

[3] A. Cassell, D. Edwards, A. Harshfield, K. Rhodes, J. Brimicombe, R. Payne and S. Griffin, "The epidemiology of multimorbidity in primary care: a retrospective cohort study," *Br J Gen Pract,* vol. 68, p. e245–e251, 2018.

[4] J. B. Schwartz, "Primary prevention: Do the very elderly require a different approach?," *Trends in Cardiovascular Medicine,* vol. 25, pp. 228-239, 2015.

[5] I. Lansdorp-Vogelaar, R. Gulati, A. B. Mariotto, C. B. Schechter, T. M. De Carvalho, A. B. Knudsen, N. T. Van Ravesteyn, E. A. M. Heijnsdijk, C. Pabiniak, M. Van Ballegooijen and others, "Personalizing age of cancer screening cessation based on comorbid conditions: model estimates of harms and benefits," *Annals of internal medicine,* vol. 161, p. 104–112, 2014.

[6] S. J. Lee, W. J. Boscardin, I. Stijacic-Cenzer, J. Conell-Price, S. O\textquoterightBrien and L. C. Walter, "Time lag to benefit after screening for breast and colorectal cancer: meta-analysis of survival data from the United States, Sweden, United Kingdom, and Denmark," *BMJ,* vol. 346, 2013.

[7] S. J. Lee, R. M. Leipzig and L. C. Walter, "Incorporating Lag Time to Benefit Into Prevention Decisions for Older Adults," *JAMA,* vol. 310, pp. 2609-2610, 12 2013.

[8] L. C. Yourman, S. J. Lee, M. A. Schonberg, E. W. Widera and A. K. Smith, "Prognostic Indices for Older Adults: A Systematic Review," *JAMA,* vol. 307, pp. 182-192, 1 2012.

[9] S. J. Lee, W. J. Boscardin, K. A. Kirby and K. E. Covinsky, "Individualizing Life Expectancy Estimates for Older Adults Using the Gompertz Law of Human Mortality," *PLOS ONE,* vol. 9, pp. 1-8, 9 2014.

[10] L. Adam, E. Moutzouri, C. Baumgartner, A. L. Loewe, M. Feller, K. M\textquoterightRabet-Bensalah, N. Schwab, S. Hossmann, C. Schneider, S. Jegerlehner, C. Floriani, A. Limacher, K. T. Jungo, C. J. A. Huibers, S. Streit, M. Schwenkglenks, M. Spruit, A. Van Dorland, J. Donzé, P. M. Kearney, P. Jüni, D. Aujesky, P. Jansen, B. Boland, O. Dalleur, S. Byrne, W. Knol, A. Spinewine, D. O\textquoterightMahony, S. Trelle and N. Rodondi, "Rationale and design of OPtimising thERapy to prevent Avoidable hospital admissions in Multimorbid older people (OPERAM): a cluster randomised controlled trial," *BMJ Open,* vol. 9, 2019.

[11] E. W. Steyerberg, K. G. M. Moons, D. A. van der Windt, J. A. Hayden, P. Perel, S. Schroter, R. D. Riley, H. Hemingway, D. G. Altman, P. R. O. G. R. E. S. S. Group and others, "Prognosis Research Strategy (PROGRESS) 3: prognostic model research," *PLoS medicine,* vol. 10, 2013.

[12] G. S. Collins, J. B. Reitsma, D. G. Altman and K. G. M. Moons, "Transparent reporting of a multivariable prediction model for individual prognosis or diagnosis (TRIPOD): the TRIPOD statement," *BMJ,* vol. 350, 2015.

[13] K. G. M. Moons, A. P. Kengne, M. Woodward, P. Royston, Y. Vergouwe, D. G. Altman and D. E. Grobbee, "Risk prediction models: I. Development, internal validation, and assessing the incremental value of a new (bio)marker," *Heart,* vol. 98, p. 683–690, 2012.

[14] K. G. M. Moons, A. P. Kengne, D. E. Grobbee, P. Royston, Y. Vergouwe, D. G. Altman and M. Woodward, "Risk prediction models: II. External validation, model updating, and impact assessment," *Heart,* vol. 98, p. 691–698, 2012.

[15] S. J. Lee, K. Lindquist, M. R. Segal and K. E. Covinsky, "Development and Validation of a Prognostic



For peer review only - http://bmjopen.bmj.com/site/about/guidelines.xhtml

Index for 4-Year Mortality in Older Adults," *JAMA,* vol. 295, pp. 801-808, 2 2006.

[16] R. D. Riley, J. Ensor, K. I. E. Snell, F. E. Harrell, G. P. Martin, J. B. Reitsma, K. G. M. Moons, G. Collins and M. van Smeden, "Calculating the sample size required for developing a clinical prediction model," *BMJ,* vol. 368, 2020.

[17] J. Ensor, E. C. Martin and R. D. Riley, "pmsampsize: Calculates the Minimum Sample Size Required for Developing a Multivariable Prediction Model," 2019.

[18] R. D. Riley, K. I. E. Snell, J. Ensor, D. L. Burke, F. E. Harrell Jr, K. G. M. Moons and G. S. Collins, "Minimum sample size for developing a multivariable prediction model: PART II - binary and time-to-event outcomes," *Statistics in Medicine,* vol. 38, pp. 1276-1296, 2019.

[19] N. R. Cook, "Use and Misuse of the Receiver Operating Characteristic Curve in Risk Prediction," *Circulation,* vol. 115, pp. 928-935, 2007.

[20] R. L. Breen, "A practical guide to focus-group research," *Journal of Geography in Higher Education,* vol. 30, p. 463–475, 2006.

[21] R. A. N. D. Corporation, "Delphi Method," [Online]. Available: https://www.rand.org/topics/delphi-method.html.

## Authors' contributions

Study concept and design: AC, NR, CDG. Acquisition, analysis, interpretation of data: NS, VG, CDG, LS. Drafting of the article: VG, CDG, AC. Critical revision of the article: VG, CDG, DA, MF, LS, NS, DB, NR, AC. Statistical analysis: VG, LS, CDG. Obtained funding: AC. Administrative, technical or material support: NS. Supervision: AC, CDG.

## Funding

This work was supported by the Swiss National Science Foundation grant number (320030_188549/01 to Prof Chiolero). This work is part of the project "OPERAM: OPtimising thERapy to prevent Avoidable hospital admissions in the Multimorbid elderly" supported by the European Union's Horizon 2020 research and innovation programme under the grant agreement No 6342388, and by the Swiss State Secretariat for Education, Research and Innovation (SERI) under contract number 15.0137. The opinions expressed and arguments employed herein are those of the authors and do not necessarily reflect the official views of the EC and the Swiss government.

## Competing interests

None declared.

## Ethics approval

The local Ethics Committee in Bern, Switzerland, approved the study protocol with the project number 2018-00784.

## Data availability statement

Data will be deposited in the Bern Open Repository and Information System (BORIS) (www.boris.unibe.ch).

## Figure legends

Figure 1: Schedule of data collection from baseline to 3-year follow-up.

Figure 2: Flowchart of the development steps (boxes) and methods used (arrows) for the life expectancy estimator tool.



For peer review only - http://bmjopen.bmj.com/site/about/guidelines.xhtml

Inclusion:
- ≥70 years
- Multimorbidity (≥3 conditions)
- Polypharmacy (≥5 drugs)

Exclusion:
- Admission to palliative care (<24 h)
- Structured drug review (<2 months)
- No informed consent

| | **Baseline**<br>First patient first visit 12/2016 | **1 year follow-up** | **2 year follow-up** | **3 year follow-up**<br>Last patient last visit 10/2021 |
|---|---|---|---|---|
| Baseline characteristics | X | | | |
| Activities of Daily Living (ADL) | X | X | | |
| Quality of Life (EQ-5D) | X | X | | |
| Falls and fractures | X | X | | |
| Health economics | X | X | | |
| Diagnoses | X | X | X | X |
| Medication | X | X | X | X |
| Blood pressure and cholesterol | X | X | X | X |
| Life expectancy questionnaire by Lee et al.[1] | | X | X | X |
| Cancer screening | | X | X | X |

[1] SJ Lee et al. "Development and Validation of a Prognostic Index for 4-Year Mortality in Older Adults". *JAMA* 2006

Schedule of data collection from baseline to 3-year follow-up.

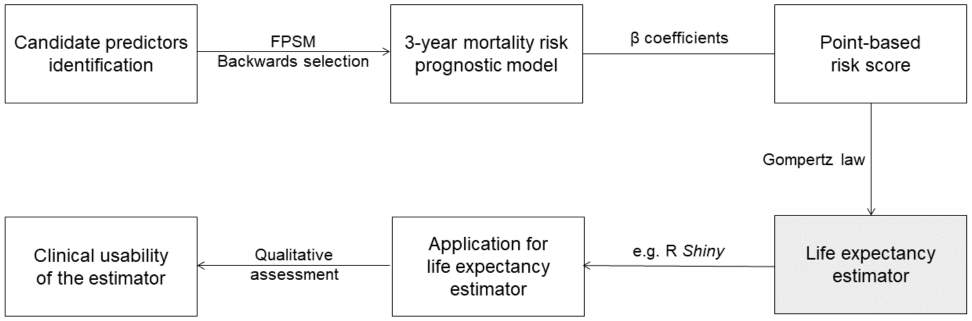


Flowchart of the development steps (boxes) and methods used (arrows) for the life expectancy estimator tool.

# TRIPOD Checklist: Prediction Model Development and Validation

| Section/Topic | Item | | Checklist Item | Page |
|---|---|---|---|---|
| **Title and abstract** | | | | |
| Title | 1 | D;V | Identify the study as developing and/or validating a multivariable prediction model, the target population, and the outcome to be predicted. | 1 |
| Abstract | 2 | D;V | Provide a summary of objectives, study design, setting, participants, sample size, predictors, outcome, statistical analysis, results, and conclusions. | 2 |
| **Introduction** | | | | |
| Background and objectives | 3a | D;V | Explain the medical context (including whether diagnostic or prognostic) and rationale for developing or validating the multivariable prediction model, including references to existing models. | 3 |
| | 3b | D;V | Specify the objectives, including whether the study describes the development or validation of the model or both. | 3 |
| **Methods** | | | | |
| Source of data | 4a | D;V | Describe the study design or source of data (e.g., randomized trial, cohort, or registry data), separately for the development and validation data sets, if applicable. | 4 |
| | 4b | D;V | Specify the key study dates, including start of accrual; end of accrual; and, if applicable, end of follow-up. | 4 |
| Participants | 5a | D;V | Specify key elements of the study setting (e.g., primary care, secondary care, general population) including number and location of centres. | 4 |
| | 5b | D;V | Describe eligibility criteria for participants. | 4 |
| | 5c | D;V | Give details of treatments received, if relevant. | 4 |
| Outcome | 6a | D;V | Clearly define the outcome that is predicted by the prediction model, including how and when assessed. | 5 |
| | 6b | D;V | Report any actions to blind assessment of the outcome to be predicted. | NA |
| Predictors | 7a | D;V | Clearly define all predictors used in developing or validating the multivariable prediction model, including how and when they were measured. | 6 |
| | 7b | D;V | Report any actions to blind assessment of predictors for the outcome and other predictors. | NA |
| Sample size | 8 | D;V | Explain how the study size was arrived at. | 7 |
| Missing data | 9 | D;V | Describe how missing data were handled (e.g., complete-case analysis, single imputation, multiple imputation) with details of any imputation method. | 7 |
| Statistical analysis methods | 10a | D | Describe how predictors were handled in the analyses. | 7 |
| | 10b | D | Specify type of model, all model-building procedures (including any predictor selection), and method for internal validation. | 7 |
| | 10c | V | For validation, describe how the predictions were calculated. | 7 |
| | 10d | D;V | Specify all measures used to assess model performance and, if relevant, to compare multiple models. | 7 |
| | 10e | V | Describe any model updating (e.g., recalibration) arising from the validation, if done. | 7 |
| Risk groups | 11 | D;V | Provide details on how risk groups were created, if done. | 7 |
| Development vs. validation | 12 | V | For validation, identify any differences from the development data in setting, eligibility criteria, outcome, and predictors. | NA |
| **Results** | | | | |
| Participants | 13a | D;V | Describe the flow of participants through the study, including the number of participants with and without the outcome and, if applicable, a summary of the follow-up time. A diagram may be helpful. | NA |
| | 13b | D;V | Describe the characteristics of the participants (basic demographics, clinical features, available predictors), including the number of participants with missing data for predictors and outcome. | NA |
| | 13c | V | For validation, show a comparison with the development data of the distribution of important variables (demographics, predictors and outcome). | NA |
| Model development | 14a | D | Specify the number of participants and outcome events in each analysis. | NA |
| | 14b | D | If done, report the unadjusted association between each candidate predictor and outcome. | NA |
| Model specification | 15a | D | Present the full prediction model to allow predictions for individuals (i.e., all regression coefficients, and model intercept or baseline survival at a given time point). | NA |
| | 15b | D | Explain how to the use the prediction model. | NA |
| Model performance | 16 | D;V | Report performance measures (with CIs) for the prediction model. | NA |
| Model-updating | 17 | V | If done, report the results from any model updating (i.e., model specification, model performance). | NA |
| **Discussion** | | | | |
| Limitations | 18 | D;V | Discuss any limitations of the study (such as nonrepresentative sample, few events per predictor, missing data). | 8 |
| Interpretation | 19a | V | For validation, discuss the results with reference to performance in the development data, and any other validation data. | NA |
| | 19b | D;V | Give an overall interpretation of the results, considering objectives, limitations, results from similar studies, and other relevant evidence. | NA |
| Implications | 20 | D;V | Discuss the potential clinical use of the model and implications for future research. | 8 |
| **Other information** | | | | |
| Supplementary information | 21 | D;V | Provide information about the availability of supplementary resources, such as study protocol, Web calculator, and data sets. | NA |
| Funding | 22 | D;V | Give the source of funding and the role of the funders for the present study. | 10 |

*Items relevant only to the development of a prediction model are denoted by D, items relating solely to a validation of a prediction model are denoted by V, and items relating to both are denoted D;V. We recommend using the TRIPOD Checklist in conjunction with the TRIPOD Explanation and Elaboration document.

For peer review only - http://bmjopen.bmj.com/site/about/guidelines.xhtml
